# Metal-centred states control carrier lifetimes in transition metal oxide photocatalysts

**DOI:** 10.1038/s41557-025-01868-y

**Published:** 2025-07-02

**Authors:** Michael Sachs, Liam Harnett-Caulfield, Ernest Pastor, Bernadette Davies, Daniel J. C. Sowood, Benjamin Moss, Andreas Kafizas, Jenny Nelson, Aron Walsh, James R. Durrant

**Affiliations:** 1 Department of Chemistry and Centre for Processable Electronics, Imperial College London, London, UK; 2 Department of Physics and Centre for Processable Electronics, Imperial College London, London, UK; 3 PULSE Institute for Ultrafast Energy Science, Stanford University, Menlo Park, CA USA; 4 Department of Materials and Centre for Processable Electronics, Imperial College London, London, UK; 5 CNRS, Univ Rennes, IPR (Institut de Physique de Rennes) - UMR 6251, Rennes, France; 6 CNRS, Univ Rennes, DYNACOM (Dynamical Control of Materials Laboratory) - IRL2015, The University of Tokyo, Tokyo, Japan

**Keywords:** Photocatalysis, Photocatalysis, Chemical physics, Energy, Crystal field theory

## Abstract

Efficient sunlight-to-energy conversion requires materials that can generate long-lived charge carriers upon illumination. However, the targeted design of semiconductors possessing intrinsically long lifetimes remains a key challenge. Here using a series of transition metal oxides, we establish a link between carrier lifetime and electronic configuration in transition metal-based semiconductors. We identify a subpicosecond relaxation mechanism via metal-centred ligand field states that compromise quantum yields in open *d*-shell transition metal oxides (for example, Fe_2_O_3_, Co_3_O_4_, Cr_2_O_3_ and NiO), which is more reminiscent of molecular complexes than crystalline semiconductors. We found that materials with spin-forbidden ligand field transitions could partially mitigate this relaxation pathway, explaining why Fe_2_O_3_ achieves higher photoelectrochemical activity than other visible light-absorbing transition metal oxides. However, achieving high yields of long-lived charges requires transition metal oxides with *d*^0^ or *d*^10^ electronic configurations (for example, TiO_2_ and BiVO_4_), where ligand field states are absent. These trends translate to transition metal-containing semiconductors beyond oxides, enabling the design of photoabsorbers with better-controlled recombination channels in photovoltaics, photocatalysis and communication devices.

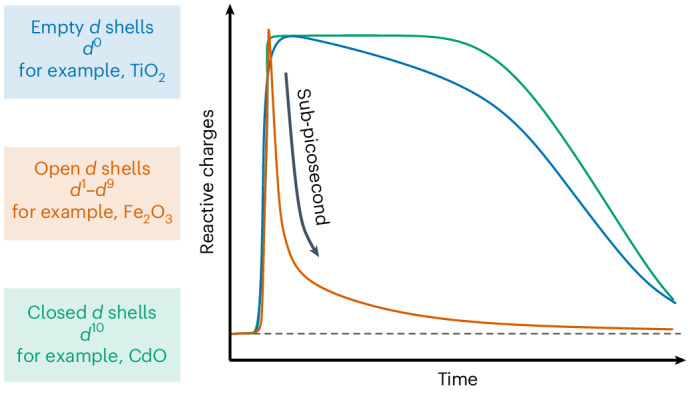

## Abstract (web summary)

Efficient sunlight-to-fuel conversion requires materials that produce long-lived charges, but increasing carrier lifetimes in visible-light-active photocatalysts has proven difficult. Now it has been shown that rapid charge deactivation via metal-centred states prevents long lifetimes and limits photocatalytic performance. These insights bridge solid-state and molecular photochemistry, informing strategies for improved photocatalyst design.

## Main

Photosynthetic devices need to couple short-lived excited states to kinetically slow chemical reactions at the catalyst surface, which requires the generation of long-lived charge carriers^1^. For example, biological photosynthesis sacrifices up to half of its light energy input to gain the carrier lifetimes required to drive chemical reactions^2^. Similarly, lifetime gain via suppression of fast recombination pathways is essential in artificial photosynthetic systems. This suppression is typically achieved through the application of external electrical bias^3,4^, the use of sacrificial reagents^5^ or the construction of rectifying junctions^6^. The design of materials with long intrinsic carrier lifetimes is key for developing more efficient solar energy conversion devices; however, it remains challenging to identify descriptors capable of predicting carrier lifetimes.

Transition metal oxides (TMOs) are the most widely studied photocatalytic materials^7^ and are of interest for low-cost photovoltaics^8^. Empirically, it has been found that photoexcitation of TMOs with open (*d*^0^) or closed (*d*^10^) *d*-shells can generate long-lived charge carriers with high solar-to-chemical conversion efficiencies^9–11^. For example, *d*^0^ oxides, such as TiO_2_ and SrTiO_3_, have reached quantum efficiencies near unity in photocatalytic applications^10,12,13^, which is correlated with longer carrier lifetimes. However, light absorption by most *d*^0^ or *d*^10^ TMOs is limited to the ultra-violet (UV) region of the solar spectrum, which constrains the amount of usable solar irradiation.

Conversely, open *d*-shell (*d*^1^–*d*^9^) TMOs absorb more visible light but struggle to efficiently convert their absorbed photons into long-lived, photocatalytically active, charges; only Fe_2_O_3_ shows appreciable yields, though still just ~34% of its maximum water oxidation photocurrent^14^. The reason for the low yields of long-lived reactive charges in open *d*-shell TMOs has remained unclear, raising the question of whether it is possible to synthesize a material with both broad spectral light absorption and high photocatalytic quantum yields in the visible range—a combination considered essential for the successful commercialization of direct solar-to-fuel devices^15^.

Here, we use time-resolved optical spectroscopy and electronic structure calculations to establish a correlation between the electronic configuration of the metal atoms in TMO photoabsorbers and the attainable carrier lifetime. We find that ligand field (LF) states, resulting from the rearrangement of the electron occupancy within the metal *d* orbitals upon photoexcitation, are responsible for a fast deactivation mechanism that ultimately sets the threshold for the attainable carrier lifetime in open *d*-shell TMOs. LF states strongly depend on the metal charge state and coordination environment and have been studied extensively for molecular complexes^16,17^. In contrast, LF states have largely been overlooked in solid-state photoabsorbers.

Our results suggest that the availability of LF states is responsible for the performance trends observed between open and empty/closed *d*-shell TMO photocatalysts. Moreover, we find that the absence of LF states also explains the success of high-absorbing, high-performing photovoltaic systems such as chalcopyrites (for example, CuInSe_2_) and halide perovskites (for example, CsPbI_3_). The model we present points towards strong parallels between TMOs and molecular light absorbers and provides a descriptor to predict and, potentially, extend carrier lifetimes in solar energy conversion materials.

## Electronic states in TMOs

**Fig. 1:**
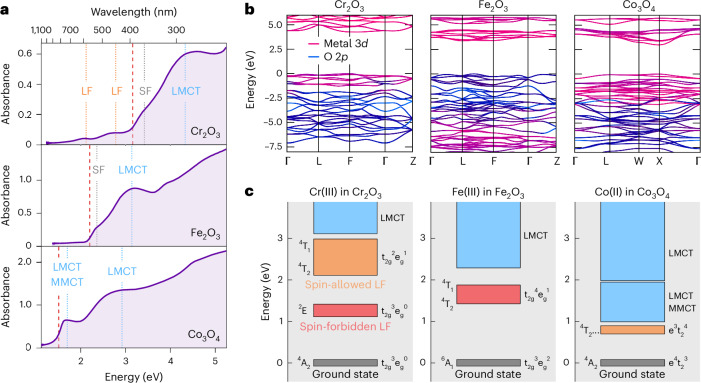
Optical and electronic properties of the open *d*-shell TMOs Cr_2_O_3_, Fe_2_O_3_ and Co_3_O_4_. **a**, Ground state optical absorption spectra in polycrystalline thin films. The dotted lines indicate LF, spin-flip (SF), LMCT and MMCT transitions. Herein, we focus on LF and LMCT states. Red dashed lines represent bandgap energies determined by Tauc plots. **b**, The single-particle electronic band structures calculated from DFT with the HSE06 exchange–correlation functional. **c**, Electronic state diagrams illustrating that LF excitations lie within the band gap of open *d*-shell TMOs, opening up pathways for energetic relaxation. Because spin changes hinder LMCT-LF transitions, we distinguish whether LF states can be accessed in a spin-allowed (orange) or spin-forbidden (red) manner from the LMCT state. The indicative optical transition energies are adapted from refs. ^18,19,22^.

To evaluate links between electronic structure and carrier lifetime, we first focus on the optical response of open *d*-shell oxides in the UV–visible (UV–vis) range, as exemplified by Fe_2_O_3_, Cr_2_O_3_ and Co_3_O_4_. Their structural characterization is shown in the Supplementary Information. The distinct absorption features in these materials (Fig. 1a) can be understood as follows (the vertical red line indicates the bandgap determined via Tauc plots in the Supplementary Information). Charge transfer (CT) transitions are optically bright with high oscillator strengths and occur from oxygen to metal (ligand-to-metal charge transfer, LMCT) or between two metal centres (metal-to-metal charge transfer, MMCT). Photoexcitation of these transitions leads directly to a degree of spatial charge separation. In contrast, LF transitions are associated with a rearrangement of electron density within the *d*-orbital manifold on the same metal centre.

Cr_2_O_3_ has a series of LF transitions at subbandgap energies due to the *d*^3^ configuration of Cr(III), which allows the absorption of visible light despite its large bandgap. The transitions around 2.1 eV (590 nm) correspond to spin-allowed t_2g_^↑↑↑^ to t_2g_^↑↑^e_g_^↑^ excitations on the Cr(III) ion in an approximately octahedral LF^18^ and contribute to the green colour of the bulk material. In contrast, subbandgap LF transitions in Fe_2_O_3_, where Fe(III) features a *d*^5^ configuration and exists within an octahedral LF, are spin-forbidden owing to the half-filled t_2g_^3^ e_g_^2^ ground state of Fe. These transitions are not well resolved owing to their low oscillator strength but can be observed from 1.4 eV (886 nm) in bulk samples^19^. The relevance of the Fe_2_O_3_ LF spin configuration for its photoelectrochemical performance is explored later. Co_3_O_4_ has CT transitions in our 0.8–2.8 eV absorption probe range, plus a combination of long-wavelength LF transitions involving tetrahedral Co(II) *d*^7^ and octahedral Co(III) *d*^6^ ions in the near-infra-red (NIR) range^20^.

The electronic composition of band states is typically rationalized using density functional theory (DFT) calculations as shown in Fig. 1b. For our open *d*-shell TMOs, O 2*p* and metal 3*d* orbitals dominate the bandgap region of the density of states. While the conduction band edge is predominantly composed of metal 3*d* states in all cases, the valence band edge exhibits increasing *O* 2*p* character from Co_3_O_4_ to Cr_2_O_3_ to Fe_2_O_3_. For comparison, for the empty *d*-shell TMO TiO_2_, the valence band edge consists almost exclusively of O 2*p* states^21^. While such orbital pictures provide insights into the CT character of a transition, they do not capture LF excitations.

LF transitions require explicit consideration of excited states that include strong excitonic effects owing to their localized nature. The energetics of atomic and molecular excitations are commonly described in state pictures, which quantify changes in energy of the entire system, in contrast to changes in orbital or band occupancy. The corresponding LF transitions can be calculated from Tanabe–Sugano diagrams. For example, the tetrahedral Co(II) ion in Co_3_O_4_ has a ^4^A_2_ ground state and a low energy ^4^T_2_ excited state, which corresponds to a change in the local electronic configuration from e^↑↓↑↓^t_2_^↑↑↑^ to e^↑↓↑^t_2_^↑↑↑↓^ (ref. ^22^).

Considering these open *d*-shell TMOs, each of the transition metals has LF states that lie at energies between the ground state and LMCT excited state (that is, at subbandgap energies) (Fig. 1c)^23^. Importantly, such LF excited states must be absent for *d*^0^/*d*^10^ oxides because their empty/filled *d* orbitals prevent the promotion of *d*-electrons within the *d*-orbital manifold. LF states are intrinsic because they are a direct consequence of a material’s electronic structure, in contrast to other intragap states such as those arising from native defects and impurities. As we explore in the following section, the presence of LF states opens up intrinsic carrier relaxation and recombination pathways, making them a key factor in solar energy conversion devices.

## Distinguishing active from inactive charges

**Fig. 2:**
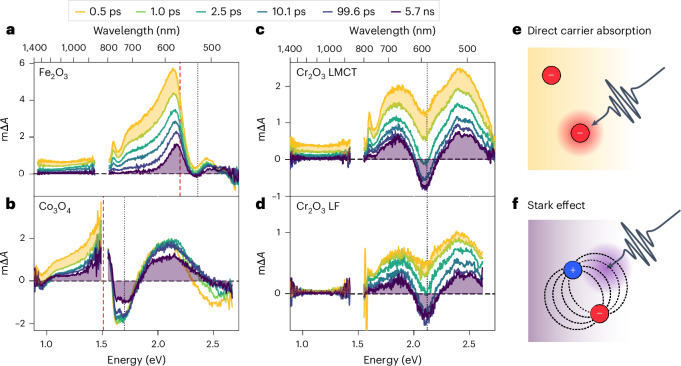
Spectral signatures of photogenerated charges on the femtosecond–nanosecond timescale. **a**–**c**, The transient absorption spectra for Fe_2_O_3_ (**a**), Co_3_O_4_ (**b**) and Cr_2_O_3_ (**c**) upon LMCT excitation (3.10 eV, 2.76 eV and 3.76 eV, respectively). **d**–**f**, The transient absorption spectra for Cr_2_O_3_ upon LF excitation (2.70 eV) (**d**). The transient absorption amplitudes are expressed as mΔ*A*, where 1 mΔ*A* = 10^–^^3^ Δ*A* (differential absorbance). The dashed black lines and dashed red lines indicate the transitions and bandgaps shown in Fig. 1a, respectively. The filled yellow and purple regions illustrate the shape of the broad and structured components discussed in the text, originating from direct carrier absorption (**e**) and the Stark effect (**f**), respectively. Note that the same number of photons absorbed was used for comparing LF and LMCT in Cr_2_O_3_. Any smaller sharp signals observed around 1.6 eV are probe light artefacts rather than real features.

To identify the nature of the photoexcited states in our TMOs, we first evaluate the femtosecond–nanosecond transient absorption spectra obtained upon LMCT excitation (Fig. 2a–c). These transient spectra consist of two signals with partial overlap: (1) a short-lived broad component observed at subbandgap energies, illustrated in yellow as the absorption difference between 0.5 and 1.0 ps, and (2) a long-lived structured component observed in spectral regions with ground state absorbance, illustrated in purple as the remaining signal after 5.7 ns. The broad component decays on the femtosecond–picosecond timescale, and is most clearly observed at NIR probe energies (Supplementary Fig. 18) where its overlap with the structured component is minimized. In contrast, the structured component has a lifetime of at least nanoseconds. As a result, the structured signal defines the overall spectral shape while the spectral evolution arises mainly from the decay of the broad component.

The broad and positive signal observed at subbandgap energies (yellow) is associated with a photoinduced optical absorption, which we assign to reactive charges that ultimately drive photocatalytic target reactions^3^. Such broad spectral signatures, often accompanied by Drude-like free carrier absorption in the infra-red range, have been observed in several *d*^0^ TMOs^24–27^. To further corroborate this assignment for open *d*-shell materials, we take advantage of the isolated LF transitions in Cr_2_O_3_, which generate spatially localized rather than mobile charges. Figure 2d demonstrates that the broad component is completely absent upon 2.7 eV (460 nm) subbandgap LF excitation, suggesting that no mobile charges are generated. Conversely, varying the excess energy of mobile charges by changing the excitation energy to different above-bandgap transitions in Co_3_O_4_ gives rise to essentially the same transient features as LMCT bandgap excitation (Supplementary Fig. 19). We thus conclude that this broad, rapidly decaying spectral feature (shaded in yellow) arises from free carriers in delocalized band-like states, as schematically shown in Fig. 2e.

We attribute the structured component (purple), observed at or near ground state absorption features, to charges trapped at defect sites. Consistent with this assignment, the amplitude of this structured signal can be modulated via the occupation of the subbandgap oxygen vacancy states in Fe_2_O_3_ (refs. ^3,28,29^) and BiVO_4_ (ref. ^30^). In a recent study, Fan et al. found that this structured signal is strongly suppressed in low-defect monocrystalline Fe_2_O_3_ films^31^, further supporting our assignment to defect states. This structured signal persists up to milliseconds and follows a power law decay at these longer times (Supplementary Fig. 20), which is indicative of a trap-mediated recombination process^32^ and consistent with a previous assignment to deeply trapped minority carriers that are largely photocatalytically inactive^28^.

The second-derivative-like shape of this structured component (Supplementary Fig. 20) suggests that it results from a broadening of the ground-state optical resonances upon photoexcitation, as exemplified by bleaches at the transition energies (dotted lines) identified in Fig. 1a. Such transition broadening in TMOs has previously been attributed to a Stark effect, caused by local internal electric fields that arise from trapped charges^27,30^ (Fig. 2f). Such second-derivative transient signals have been linked to trapped charges in transition metal selenides^33,34^ and halide perovskites^35,36^, as well as to other forms of localized carriers^37^. We explore these less functionally relevant trapped carriers in the Supplementary Information, shifting our focus to the more mobile and reactive charges for the remainder of this paper.

## Temporal evolution of reactive charges

**Fig. 3:**
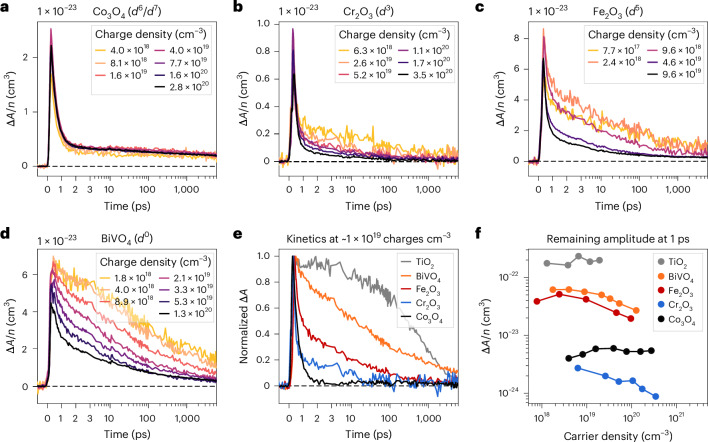
Dynamics of photogenerated charges on the femtosecond–nanosecond timescale. **a**–**d**, The transient absorption kinetics probed at 1.13 eV (1,100 nm) as a function of photogenerated charge density for Co_3_O_4_ (1.68 eV excitation) (**a**), Cr_2_O_3_ (3.40 eV excitation) (**b**), Fe_2_O_3_ (3.10 eV excitation) (**c**) and BiVO_4_ (3.54 eV excitation) (**d**). The signal amplitude is reported as Δ*A*/*n*, where the transient absorption difference signal Δ*A* is divided by the photogenerated charge density *n*, to more easily distinguish between charge-density-independent (monomolecular) and charge-density-dependent (for example, bimolecular) loss processes. **e**, Comparison of the broad component kinetics seen in Co_3_O_4_, Cr_2_O_3_, Fe_2_O_3_ and BiVO_4_ obtained from low fluence measurements (~1 × 10^19^ charges cm^−3^), where any slowly decaying background arising from a structured component has been subtracted. Analogous kinetics for TiO_2_ anatase are included for comparison (ref. ^38^). Traces have been normalized to the signal maximum. **f**, The remaining signal fraction at 1 ps as a function of carrier density, as an indication of the yield of charge separation (but note differences in absorption coefficients may also contribute to differences in signal intensity).

To capture the temporal evolution of reactive charge populations, we turn to the kinetics of the broad component in our transient signal, assigned to carriers in band-like states as detailed earlier. Figure 3 shows transient kinetics probed at subbandgap energies for different fluences, representing decays at different charge carrier densities. Signal amplitudes are represented as Δ*A*/*n*, where the transient absorption difference signal Δ*A* is divided by the photogenerated charge density *n*. Using this Δ*A*/*n* metric, overlapping traces reveal charge-density-independent (that is, monomolecular) processes. In contrast, a divergence between traces suggests a charge-density-dependent process involving more than one charge, a common example of which is bimolecular electron–hole recombination.

The Co_3_O_4_ kinetics shown in Fig. 3a exhibit two charge-density-independent kinetic processes, namely, a rapid exponential decay with a time constant of 400 fs and a small slowly decaying background. The slowly decaying background in Co_3_O_4_ captures the slow decay of its structured component. Because the structured component (Fig. 2a–d, purple shaded area) is not probed for any of the other oxides presented in Fig. 3 at the chosen probe energy of 1.13 eV, we do not address it further in the following discussion of reactive charge dynamics. The subpicosecond decay in Co_3_O_4_ is associated with the disappearance of the broad component, suggesting a rapid monomolecular decay of reactive charges, almost irrespective of the charge carrier density studied. However, for Cr_2_O_3_ (Fig. 3b) and Fe_2_O_3_ (Fig. 3c), in addition to this subpicosecond decay, we observe a broad charge-density-dependent component that decays largely on the picosecond–nanosecond timescale. The decay of this second population accelerates with increasing charge density, which is indicative of the bimolecular recombination of separated electrons and holes, and is consistent with our assignment of this spectral feature to mobile, reactive charges. For comparison, BiVO_4_, a *d*^0^ metal oxide, primarily exhibits bimolecular recombination kinetics (Fig. 3d).

It is apparent that the lifetime of photogenerated charges strongly varies between oxides. Figure 3e compares the kinetics of reactive charges seen at low fluences in Fig. 3a–c across oxides, for which we subtract the structured component (Supplementary Fig. 24). The subpicosecond monomolecular decay is observed for all of our open *d*-shell oxides, which we attribute to a loss of reactive charges via relaxation through their LF states, illustrated in Fig. 1c. This LF relaxation process is temperature independent and thus barrierless (Supplementary Fig. 23). Data reported by Fan et al.^31^ further demonstrate that this LF relaxation process is active even in highly crystalline samples, supporting its materials-intrinsic nature. In *d*^0^ and *d*^10^ TMOs, where LF states are absent, this relaxation pathway is suppressed and charges undergo slower bimolecular recombination, as illustrated for BiVO_4_ and TiO_2_ anatase^38^. As a result, with the partial exception of Fe_2_O_3_, a substantially larger proportion of the signal decays within the first picosecond in open *d*-shell TMOs compared with those with *d*^0^ configurations (Fig. 3f).

Furthermore, our data suggest that differences in energy and spin between the LMCT and LF states in open *d*-shell TMOs control the dominance of LF relaxation. The dynamics of Co_3_O_4_ are strongly dominated by LF relaxation, in line with a small LMCT-LF energy gap and a spin-allowed LMCT-LF transition. In contrast, Fe_2_O_3_ exhibits a larger population of longer-lived, reactive charges than the other open *d*-shell metal oxides (Fig. 3f), which can be primarily attributed to its LMCT-LF transition being spin-forbidden (Fig. 1c, red LF states). In addition, lower electron–phonon coupling in Fe_2_O_3_ may play a role (Supplementary Information). This larger population of reactive charges explains the comparably higher photoelectrochemical performance of Fe_2_O_3_ for an open *d*-shell TMO.

## Correlating electronic configuration and carrier lifetime

We now extend our observations to a wider range of TMOs. We first consider the effects of bandgap size and doping density on carrier lifetime. In NiO, Ni(II) has a *d*^8^ configuration with LF transitions at 1.1, 1.8 and 3.2 eV (ref. ^23^). For an open *d*-shell TMO, NiO has a large bandgap of ca. 3.8 eV yet exhibits fast monomolecular recombination (Supplementary Fig. 25), demonstrating that it is not the typically smaller bandgap of open *d*-shell TMOs that causes higher recombination rates, as might have been expected on the basis of the energy gap law^39^.

Conversely, CdO is a closed *d*-shell *d*^10^ TMO with an optical bandgap similar to Fe_2_O_3_, yet it exhibits long-lived mobile charges (Supplementary Fig. 26). Mobile charges in *d*^10^ TMOs appear in the form of a near-band edge bleach, similar to widely used photovoltaic materials, such as lead halide perovskites (for example, MAPI)^40^ or Cu(In,Ga)Se_2_ (ref. ^41^), which incorporate Pb(II) and Cu(I) *d*^10^ metal centres, respectively. At the other end of the Periodic Table, *d*^0^ oxides, such as BiVO_4_ and anatase TiO_2_, exhibit bimolecular behaviour and slower decays than open *d*-shell oxides even in materials with large intrinsic doping densities (rutile TiO_2_ and WO_3_) (refs. ^38,42^).

**Fig. 4:**
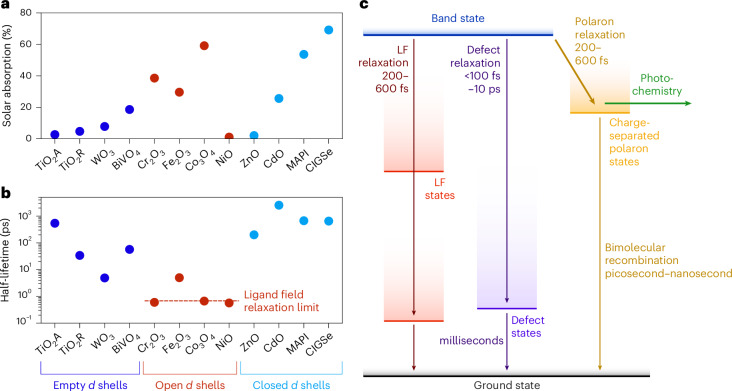
Effect of electronic configuration on light absorption and carrier lifetime. **a**, The light harvesting potential in various transition metal-containing light absorbers measured as a percentage of absorbed solar power for an AM 1.5 solar spectrum, assuming full absorption of photons with an energy above the absorption onset. **b**, The half-lifetime of reactive charges at the lowest used fluence, including literature values for TiO_2_ (ref. ^38^), WO_3_ (ref. ^42^), ZnO (ref. ^43^), MAPI^40^ and CIGSe^41^. **c**, The electronic state diagram summarizing the possible localization and recombination processes upon photoexcitation. We note that defect states can also contribute to LF relaxation and polaron recombination.

In open *d*-shell TMOs, *d* states incorporate into the valence band and/or introduce lower energy LF transitions, which can give rise to more extended visible light absorption. By contrast, TMOs with *d*^0^ or *d*^10^ configurations tend to absorb a relatively small fraction of solar photons owing to their larger bandgaps (Fig. 4a). At the same time, the availability of LF states associated with partially occupied *d* orbitals induces a pathway for rapid relaxation of LMCT states, leading to much shorter overall lifetimes of reactive charges (Fig. 4b). Interestingly, certain *d*^10^ materials, including CdO as well as the lead halide perovskite and chalcogenide semiconductors primarily used in solar cells (for example, MAPI and CIGSe), break this anticorrelation and offer both extended light absorption and long carrier lifetimes. These examples illustrate that *d*^10^ materials are particularly promising candidates to develop visible light absorbers with long carrier lifetimes and that our lifetime model generalizes to other semiconductors beyond oxides.

## Competing charge relaxation pathways

Our results point to rapid relaxation through LF states being the primary reason for the insufficient activity of open *d*-shell photocatalysts—a pathway that, until now, has received little attention for solid-state materials. LF relaxation is non-radiative, consistent with the negligible photoluminescence of open *d*-shell TMOs, and is most pronounced for spin and parity-allowed transitions (for example, in Co_3_O_4_). By combining the findings presented herein with the existing literature^38,40–43^, we establish a generalized photophysical model of TMO semiconductors, as illustrated in Fig. 4c. Upon bandgap photoexcitation, relatively delocalized but short-lived band-like states are generated, which subsequently evolve via one of three pathways: (1) LF states promote the subpicosecond relaxation of these band-like charges in open *d*-shell TMOs, (2) minority carrier trapping occurs in spatial proximity to physical defects, predominantly on the subpicosecond timescale and (3) band-like charges localize as spatially separated polarons that subsequently recombine bimolecularly on the picosecond–nanosecond timescale and can drive chemical reactions on longer timescales, for example, in the presence of electrical bias.

An energy cascade is created if the energy gaps between LF states are sufficiently small, supporting fast relaxation through non-radiative dissipation of energy via electron–phonon coupling. The prominence of LF relaxation is a material property, in line with the associated decay being independent of applied potential^3,44^ or defect concentration^31^. LF relaxation is driven by the electronic configuration of the metal centre, consistent with an analogous fast relaxation pathway being observed for a number of solvated molecules that incorporate open *d*-shell transition metal centres, where fast intersystem crossing followed by rapid internal conversion through LF states leads to short excited state lifetimes^45,46^. LF relaxation in open *d*-shell TMOs is also in line with an observed lower yield of mobile carriers in Fe_2_O_3_ compared with TiO_2_ (ref. ^47^), and phenomenologically similar to carrier relaxation though high densities of defect states in defect-rich WO_3_ (ref. ^42^).

A subpicosecond formation of small polarons in Fe_2_O_3_ has been inferred using transient extreme UV spectroscopy^48–51^ and pump–push photocurrent detection^44^, with comparably fast polaron formation dynamics being observed in materials such as α-FeOOH (ref. ^52^), CeO_2_ (ref. ^53^) and NiO (ref. ^54^). Our observation of LF relaxation on the same timescale suggests a kinetic competition between these processes. The resulting polaron population defines the number of potentially active charges and is therefore a central descriptor for activity, as supported by studies on n-type TMOs where the application of positive potential slows down^3,55,56^ the bimolecular recombination of polarons and gives rise to long-lived reactive holes.

Polaron formation occurs with a non-radiative energy loss^57–60^, and the resultant lower mobility of polarons compared with more band-like charges is largely considered to be detrimental to electronic device function. However, on the basis of a comparison of the different relaxation timescales (Fig. 4c), we speculate that ultrafast polaron formation may present a way to mitigate rapid (subpicosecond) LF relaxation in open *d*-shell TMOs by stabilizing a spatial separation of localized charges. While further work is needed to establish the role of polarons in mitigating LF relaxation in TMOs, similar localization-induced charge separation has been suggested to be important in organic solar cells, analogous to Onsager models of auto-ionization^61^.

In the presence of structural defects, such as oxygen vacancies, carrier localization can also occur via trapping of minority charges. If the associated trap states are energetically deep, they can render the localized charge inactive (Supplementary Information). Minority carrier trapping in open *d*-shell TMOs has been observed to take place mainly from the more delocalized band states on a sub-100-fs timescale^28^, although it can extend out to 10 ps in BiVO_4_ (ref. ^30^). Once polarons form, their probability of encountering a physical defect over their lifetime decreases owing to their more confined wavefunctions, enabling longer polaron diffusion lengths in some cases^62^.

## Implications for photocatalyst design

While our results suggest that LF relaxation is insensitive to applied potential, defect concentration or temperature, we highlight two distinct strategies for improving solar-to-energy conversion efficiencies. The first is to develop *d*^0^ and *d*^10^ metal oxides with extended visible light absorption, which prevent LF relaxation owing to the absence of LF states in materials with empty or closed *d* shells. Several promising photocatalysts already combine *d*^0^/*d*^10^ configurations with visible light absorption (for example, ZnGaON, CdS, TaON and Ta_3_N_5_), and the need for open/closed *d*-shell configurations has been recognized empirically^63–65^. Moreover, our observations are in good agreement with the lower conductivity of open *d*-shell TMOs, which has been rationalized by considering the more localized nature of the band edges in these materials^66,67^. The second strategy is to engineer new open *d*-shell TMOs in which the effect of LF states can be controlled, which leverages the typically better visible light absorption of these materials while mitigating the detrimental effects of LF relaxation.

While choosing materials with empty or closed *d*-shell configurations is relatively straightforward, controlling LF states in open *d*-shell photoabsorbers is challenging. However, the lessons learned for transition metal-based molecular complexes point to possible directions for controlling LF states^68–70^. A key strategy in these molecular complexes is to increase the strength of the LF, which can increase the energy of LF states above that of CT states (LMCT, MLCT or band states), thus making LF state-mediated relaxation energetically unfavourable^70^. The strength of the LF can be modified by tuning the central metal or its ligand environment.

In terms of metal selection, the 4*d* or 5*d* orbitals of second- or third-row metals are less spatially contracted than the 3*d* orbitals of first-row transition metals, which leads to larger metal–ligand overlap and therefore a stronger LF for 4*d* or 5*d* metals, shifting LF states to higher energies. For example, [Fe(bpy)_3_]^2+^, [Ru(bpy)_3_]^2+^ and [Os(bpy)_3_]^2+^ are isoelectronic and thus have the same electronic states, but only [Fe(bpy)_3_]^2+^ exhibits LF relaxation because its lowest energy excited state is an LF state^69^.

In terms of the ligand environment, strong *σ* donors or strong *π* acceptors facilitate strong LFs by increasing the energy of the e_g_ orbitals or lowering that of the t_2g_ orbitals, respectively^70^. For example, while the lifetime of [Fe(bpy)_3_]^2+^ is ~100 fs owing to rapid LF relaxation, coordination of Fe complexes with tris-carbene ligands can achieve lifetimes on the nanosecond timescale and enable photochemistry. The strongly σ-donating carbene ligands promote a large LF that shifts low energy LF states to energies above the lowest-lying LMCT state^71^.

Applying these insights to TMOs, 4*d* or 5*d* metals can be advantageous owing to their more diffuse *d* orbitals, which enhance the metal–oxygen orbital overlap. However, semiconducting properties are rare among binary oxides of these metals, suggesting a look towards halide- or pnictide-containing materials. The O^2−^ ‘ligands’ in TMOs are strong *σ* donors but lack *π* acceptor ability, which leads to an increased e_g_ orbital energy and a moderately strong LF. To further enhance LF strength, increasing the metal oxidation state can be effective, enhancing metal–oxygen orbital overlap and covalency. Lattice compression can achieve similar effects by reducing metal–ligand bond lengths, which can be accomplished through Jahn–Teller distortions or strain engineering in thin films.

For further increases in LF strength, moving to more complex materials may be beneficial. For example, oxide perovskites offer flexible crystal structures that allow for greater metal–oxygen hybridization. Moreover, beyond lifetime control, larger *d* orbitals and stronger covalent interactions that give rise to broader bands that can mitigate the poor electrical conductivity of Mott insulators. Ultimately, the design of high-performance photocatalysts is a challenging task that requires a balance between multiple factors such as carrier lifetime, band alignment and conductivity.

## Conclusions

In this work, we identify LF states as the critical determinant of ultrafast charge carrier deactivation in open *d*-shell TMOs. Our data reveal a rapid subpicosecond decay of reactive charges, consistent with relaxation through LF states that limits the yield of the long-lived carriers that are essential for efficient photocatalysis. We argue that this intrinsic relaxation mechanism, largely overlooked in solid-state semiconductors, substantially diminishes the photocatalytic quantum yields of open *d*-shell oxides such as Fe_2_O_3_, Co_3_O_4_ and Cr_2_O_3_. Notably, the spin-forbidden LMCT-to-LF transition in Fe_2_O_3_ results in comparatively higher yields of long-lived charges than other open *d*-shell TMOs, although still lower than UV absorbing *d*^0^/*d*^10^ materials.

We establish that the absence of LF states in *d*^0^ and *d*^10^ semiconductors inherently favours longer carrier lifetimes, explaining the superior quantum efficiencies of *d*^0^/*d*^10^ transition metal-based semiconductors in photocatalysis (for example, SrTiO_3_, TiO_2_ and BiVO_4_) and in photovoltaics (for example, MAPI and CIGSe materials). Given the critical importance of electronic configurations in determining solar energy conversion efficiencies, we discuss two complementary strategies towards next-generation photocatalysts: developing visible light-absorbing *d*^0^/*d*^10^ oxides or engineering LFs in open *d*-shell TMOs to mitigate LF-mediated recombination. Our lifetime-based model bridges solid-state and molecular photochemistry, offering a unified design framework to rationally tune the excited state lifetimes of transition metal-based semiconductors for improved performance in photocatalysis, photovoltaics and communication devices.

## Methods

### Thin film preparation

The studied metal oxides were prepared via sol–gel synthesis on quartz glass substrates. The following synthetic protocols are adapted or reproduced here from ref. ^72^.

#### Cr_2_O_3_

Cr(NO_3_)_3_·9H_2_O (0.25 g, 0.06 mmol) and propylene oxide 469 μl were dissolved in ethanol (2.5 ml). The solution was stirred at 60 °C for 4 h and then taken off the hotplate. Rapid gelation started to occur after 30 min of resting. The gelating solution was spin coated onto quartz glass substrates (4,000 rpm for 1 min), yielding a series of films with different thicknesses that were then annealed in air (15 min to 80 °C, 30 min at 80 °C, 1 h to 500 °C and 3 h at 500 °C). The film with the most appropriate thickness was then selected after the preparation.

#### Fe_2_O_3_

Fe_2_O_3_ thin films were prepared on the basis of a previously reported procedure^73^. FeCl_2_·4H_2_O (0.42 g, 0.33 mmol), citric acid (0.64 g, 0.33 mmol) and ascorbic acid (0.059 g, 0.33 mmol) were dissolved in N_2_-saturated ethanol (10 ml), where citric acid acts as a chelating agent and ascorbic acid is an antioxidant. The resulting solution was stirred in a closed round-bottom flask at 60 °C for 5–6 h. After this time, 20 μl of dimethylformamide was added as a drying control reagent and the solution was stirred for an additional 30 min. The solution was then spin coated onto quartz glass substrates (5,000 rpm for 1 min), and the deposited films were annealed in air (20 min to 300 °C and 30 min at 300 °C). A second layer was then spin coated on top of the first one (5,000 rpm for 1 min), and the films were annealed again (20 min to 300 °C and 30 min at 300 °C). Finally, the films were annealed at 450 °C for 3 h to complete the conversion to α-Fe_2_O_3_.

#### Co_3_O_4_

Co_3_O_4_ thin films were prepared on the basis of a previously reported procedure^74^. Co(NO_3_)_2_·6H_2_O (0.50 g, 1.72 mmol) was dissolved in ethanol (2.5 ml). A second solution was prepared by dissolving ethylcellulose (0.125 g) and α-terpineol (2.35 g, 15.3 mmol) and was eventually decanted since ethylcellulose did not fully dissolve. Both solutions were then mixed inside a round-bottom flask and stirred for 30 min at room temperature. The solution was then spin coated onto quartz glass substrates (2,000 rpm for 1 min), and the deposited films were annealed in air (15 min to 100 °C, 15 min at 100 °C, 30 min to 500 °C and 1 h at 500 °C).

#### BiVO_4_

BiVO_4_ thin films were prepared on the basis of a previously reported procedure^56,75,76^. Bismuth nitrate pentahydrate (0.1455 g, 200 mM) was dissolved in acetic acid (1.5 ml), and vanadyl acetyl acetone (0.0768 g, 30 mM) was dissolved in acetyl acetone (10 ml). Subsequently, these two solutions were mixed and stirred at room temperature for 30 min. The resulting sol–gel mixture was then spin coated onto quartz glass substrates, and the substrates were annealed at 450 °C for 10 min. This process was repeated three more times to reach a total of four layers. After the deposition of the final layer, the films were annealed at 450 °C for 5 h in air.

#### NiO

Nickel acetate tetrahydrate (498 mg, 2 mmol) and ethanolamine (240 μl) were dissolved in 2-methoxyethanol (3 ml). The solution turned deep blue in colour immediately upon addition of ethanolamine and was then stirred at 100 °C for 30 min. The solution was then spin coated onto quartz glass substrates (4,000 rpm for 40 s), and the deposited films were annealed at 350 °C for 3 h in air.

#### CdO

Cadmium acetate (461 mg, 2 mmol) and ethanolamine (120 μl) were dissolved in 2-methoxyethanol (2 ml) and stirred at room temperature overnight (∼15 h). The solution was then spin coated onto quartz glass substrates (5,000 rpm for 1 min), and the deposited films were annealed in air (40 min to 130 °C, 20 min at 130 °C, 1.5 h to 500 °C and 4 h at 500 °C).

### X-ray diffraction

Details of the x-ray diffraction setup and analysis routine can be found in ref. ^77^. In brief, x-ray diffraction measurements were performed using an x-ray diffractometer (Bruker D2 Phaser, equipped with parallel beam optics and a Lynx-Eye detector). A Cu source (*V* = 30 kV, *I* = 10 mA) was used, producing K_α1_ (*λ* = 1.54056 Å) and K_α2_ (*λ* = 1.54439 Å) x-rays in an intensity ratio of 2:1. The collected diffraction data were compared with standards on the Physical Sciences Data-Science database. The lattice parameters were determined using the Le Bail model, and the average crystallite size was calculated using the Scherrer relationship.

### Raman spectroscopy

The Raman spectra for each thin film were measured using one of two Raman spectrometers (Renishaw 1000, equipped with a 633-nm laser and Bruker Senterra II, equipped with a 532-nm laser). The measured Raman spectra were compared with published literature data, extracted from their respective publications using a plot digitizer.

### Absorption spectroscopy

Absorbance measurements were carried out using a UV–vis spectrometer (Agilent Cary 60).

### Transient absorption spectroscopy (femtosecond–nanosecond timescale)

The following description is adapted from ref. ^72^, where additional details can be found. The ultrafast transient absorption setup uses a regeneratively amplified Ti:sapphire laser (Solstice, Spectra-Physics), which produces 800 nm laser pulses with a width of 92 fs at a 1 kHz repetition rate. The transient absorption setup is commercially available (Helios, Ultrafast Systems). After the Solstice amplifier, each pulse is divided into what will become pump and probe pulses using a semi-transparent mirror. The pump pulse is directed through an optical parametric amplifier (TOPAS Prime, Light Conversion) and a frequency mixer (NirUVis, Light Conversion), which allows for tuning of the excitation wavelength from ∼290 nm into the NIR region. The probe pulse is directed through a delay stage, which delays it by an adjustable time period with respect to its corresponding pump pulse, thus defining the time at which the sample is probed. The maximum delay is ultimately defined by the total length of the delay stage and in this case corresponds to ∼6 ns. After the delay stage, the (at this point still 800 nm) probe pulse is focussed into a sapphire crystal, which transforms the monochromatic beam into a white light continuum via self-phase modulation. The resulting continuum allows for an entire wavelength range to be probed at once. Depending on the thickness of the inserted sapphire crystal, either a visible probe continuum (450–800 nm) or an NIR probe continuum (850–1,400 nm) can be generated. The generated continuum pulses are then again divided using a semi-transparent mirror, where one of them probes the sample and the other one serves as a reference to account for fluctuations and improve the signal-to-noise ratio. Each of the two continuum pulses is eventually focussed into a separate multichannel spectrometer (Si or InGaAs sensors) via optical fibres. The continuum pulse that probes the sample is then spatially overlapped with the pump pulse on the sample. The transient absorption signal Δ*A* is calculated as *A*_ES_ − *A*_GS_, where *A*_ES_ is the sample absorbance in the excited state and *A*_GS_ is the sample absorbance in the ground state. The measurement of *A*_ES_ and *A*_GS_ is achieved by blocking every other pump pulse with an optical chopper rotating at 500 Hz. Pulse energies were measured using an energy meter (VEGA P/N 7Z01560, OPHIR Photonics) equipped with a 500-μm diameter aperture, roughly corresponding to the diameter of the probe beam. The pump beam was slightly larger than 500 μm at the sample position. During data collection, samples were kept under continuous nitrogen flow in a quartz cuvette.

### Transient absorption spectroscopy (microsecond–second timescale)

The following description is adapted from ref. ^72^, where additional details can be found. Transient absorption experiments on the microsecond–second timescale were performed using a home-built setup. The microsecond–second TAS setup uses a Nd:YAG laser (OPOTEK Opolette 355 II, 4–7 ns pulse width), which generates UV excitation pulses (355 nm, fixed) via a third harmonic output port as well visible/NIR pulses (410–2,200 nm, tuneable) via an optical parametric oscillator. The selected pump pulses are then directed to the sample via a liquid light guide, and are overlapped with the probe beam at the position of the sample. The probe beam originates from a 100-W quartz halogen lamp driven by a stabilized power supply (Bantham IL1), which is sequentially directed through a first monochromator, the sample, and then a second monochromator before it impinges onto a Si photodiode detector (Hamamatsu S3071). Additional appropriate long pass filters are positioned between the sample and the detector to attenuate scattered laser light. Data acquisitions are triggered by a scattered laser light using a photodiode (Thorlabs DET210) positioned close to the laser output. A home-built LabVIEW-based software package acquires data on two different time scales simultaneously: the microsecond–millisecond signal is sampled using an oscilloscope (Tektronix DPO 2012B) after amplification (Costronics 1999 amplifier), whereas the millisecond–second signal is sampled without amplification using a data acquisition card (National Instruments USB-6211 or USB-6361). Excitation fluences were measured using a pyroelectric energy sensor (Ophir Photonics PE9). During data collection, samples were kept under continuous nitrogen flow in a quartz cuvette.

### Temperature-dependent absorption and transient absorption experiments

For experiments as a function of temperature, the studied thin film sample was placed in an optical cryostat (Oxford Instruments OptistatDN-V), which can access a temperature range of 77–500 K. The cryostat was then inserted at the sample position of the UV–vis spectrometer or transient absorption setup. During the temperature-dependent experiments, the samples were kept under vacuum in the cryostat.

### Electronic structure calculations

The conventional crystallographic unit cells with magnetic data for the three oxide compounds (Fe_2_O_3_, Cr_2_O_3_ and Co_3_O_4_) were obtained from the MAGNDATA database^78^. The shape and internal parameters of each unit cell were optimized on the basis of the forces and stresses from DFT, as implemented in the Vienna Ab Initio Simulation Package. A 550 eV plane wave cut-off energy was used in all cases to define the basis set, and spin-unrestricted calculations were performed, including scalar-relativistic correctors through the projector augmented wave method. The resulting energy versus volume curve, obtained using the PBEsol semi-local exchange–correlation functional, was fitted to the Murnaghan equation of state to deduce the optimal unit cell volume. A 3 × 3 × 3 Monkhorst–Pack mesh was used to sample the first Brillouin zone for Co_3_O_4_, while a 5 × 5 × 2 Monkhorst–Pack mesh was used for Cr_2_O_3_ and Fe_2_O_3_.

A 5 × 5 × 5 **k**-point grid was used for the electronic minimization of the primitive unit cells for each oxide compound. The ‘sumo-kgen’ code^79^ was used to generate respective band structure paths in reciprocal space for each compound. These **k**-point paths and their corresponding input files were then used to perform the band structure calculations using the HSE06 hybrid exchange-correlation functional. The ‘sumo-bandplot’ code^80^ was used to analyse the output. The Co_3_O_4_ path contained 56 **k**-points, while the Fe_2_O_3_ and Cr_2_O_3_ paths contained 109 **k**-points. The paths were split into smaller subjobs and then concatenated to reduce the computational overhead.

## Online content

Any methods, additional references, Nature Portfolio reporting summaries, source data, extended data, supplementary information, acknowledgements, peer review information; details of author contributions and competing interests; and statements of data and code availability are available at 10.1038/s41557-025-01868-y.

## Supplementary information

Supplementary Information Supplementary Figs. 1–26, Discussion and Tables 1 and 2.

## Data Availability

The datasets underlying the figures presented in the main paper and Supplementary Information are available via Zenodo at 10.5281/zenodo.15198620 (ref. ^81^).

## References

[1] Godin, R., Kafizas, A. & Durrant, J. R. Electron transfer dynamics in fuel producing photosystems. *Curr. Opin. Electrochem.* **2**, 136–143 (2017).

[2] Godin, R. & Durrant, J. R. Dynamics of photoconversion processes: the energetic cost of lifetime gain in photosynthetic and photovoltaic systems. *Chem. Soc. Rev.* **50**, 13372–13409 (2021). 34786578 10.1039/d1cs00577d

[3] Pendlebury, S. R. et al. Ultrafast charge carrier recombination and trapping in hematite photoanodes under applied bias. *J. Am. Chem. Soc.* **136**, 9854–9857 (2014). 24950057 10.1021/ja504473e PMC4210134

[4] Cowan, A. J., Tang, J., Leng, W., Durrant, J. R. & Klug, D. R. Water splitting by nanocrystalline TiO_2_ in a complete photoelectrochemical cell exhibits efficiencies limited by charge recombination. *J. Phys. Chem. C* **114**, 4208–4214 (2010).

[5] Pellegrin, Y. & Odobel, F. Sacrificial electron donor reagents for solar fuel production. *C. R. Chim.* **20**, 283–295 (2017).

[6] Moniz, S. J. A., Shevlin, S. A., Martin, D. J., Guo, Z.-X. & Tang, J. Visible-light driven heterojunction photocatalysts for water splitting—a critical review. *Energy Env. Sci* **8**, 731–759 (2015).

[7] Fujishima, A. & Honda, K. Electrochemical photolysis of water at a semiconductor electrode. *Nature* **238**, 37–38 (1972). 12635268 10.1038/238037a0

[8] Rühle, S. et al. All-oxide photovoltaics. *J. Phys. Chem. Lett.* **3**, 3755–3764 (2012). 26291107 10.1021/jz3017039

[9] Wang, Q., et al. Scalable water splitting on particulate photocatalyst sheets with a solar-to-hydrogen energy conversion efficiency exceeding 1%. *Nat. Mater*. 10.1038/nmat4589 (2016). 10.1038/nmat4589 26950596

[10] Takata, T. et al. Photocatalytic water splitting with a quantum efficiency of almost unity. *Nature* **581**, 411–414 (2020). 32461647 10.1038/s41586-020-2278-9

[11] Wang, S. et al. New BiVO4 dual photoanodes with enriched oxygen vacancies for efficient solar-driven water splitting. *Adv. Mater.* **30**, 1800486 (2018). 10.1002/adma.201800486 29602201

[12] Liu, M., de Snapp, N. L. & Park, H. Water photolysis with a cross-linked titanium dioxide nanowire anode. *Chem. Sci.* **2**, 80–87 (2010).

[13] Wilson, A. A. et al. Transient absorption spectroscopy reveals that slow bimolecular recombination in SrTiO_3_ underpins its efficient photocatalytic performance. *Chem. Commun.* **59**, 13579–13582 (2023). 10.1039/d3cc04616h 37905723

[14] Kim, J. Y. et al. Single-crystalline, wormlike hematite photoanodes for efficient solar water splitting. *Sci. Rep.* **3**, 2681 (2013). 24045290 10.1038/srep02681 PMC3775410

[15] Chen, S., Takata, T. & Domen, K. Particulate photocatalysts for overall water splitting. *Nat. Rev. Mater* **2**, 17050 (2017).

[16] Zhang, W. & Gaffney, K. J. Mechanistic studies of photoinduced spin crossover and electron transfer in inorganic complexes. *Acc. Chem. Res.* **48**, 1140–1148 (2015). 25789406 10.1021/ar500407p

[17] Wegeberg, C. & Wenger, O. S. Luminescent first-row transition metal complexes. *JACS Au* **1**, 1860–1876 (2021). 34841405 10.1021/jacsau.1c00353 PMC8611671

[18] McClure, D. S. Comparison of the crystal fields and optical spectra of Cr_2_O_3_ and ruby. *J. Chem. Phys.* **38**, 2289–2294 (1963).

[19] Marusak, L. A., Messier, R. & White, W. B. Optical absorption spectrum of hematite, αFe_2_O_3_ near IR to UV. *J. Phys. Chem. Solids* **41**, 981–984 (1980).

[20] Waegele, M. M., Doan, H. Q. & Cuk, T. Long-lived photoexcited carrier dynamics of *d*–*d* excitations in spinel ordered Co_3_O_4_. *J. Phys. Chem. C* **118**, 3426–3432 (2014).

[21] Scanlon, D. O. et al. Band alignment of rutile and anatase TiO_2_. *Nat. Mater.* **12**, 798–801 (2013). 23832124 10.1038/nmat3697

[22] Miedzinska, K. M. E., Hollebone, B. R. & Cook, J. G. An assignment of the optical absorption spectrum of mixed valence Co_3_O_4_ spinel films. *J. Phys. Chem. Solids* **48**, 649–656 (1987).

[23] Cox, P. A. *Transition Metal Oxides: an Introduction to Their Electronic Structure and Properties* (Clarendon, 2010).

[24] Yoshihara, T. et al. Identification of reactive species in photoexcited nanocrystalline TiO_2_ films by wide-wavelength-range (400–2500 nm) transient absorption spectroscopy. *J. Phys. Chem. B* **108**, 3817–3823 (2004).

[25] Yamada, Y., Yasuda, H., Tayagaki, T. & Kanemitsu, Y. Photocarrier recombination dynamics in highly excited SrTiO_3_ studied by transient absorption and photoluminescence spectroscopy. *Appl. Phys. Lett.* **95**, 121112 (2009).

[26] Tamaki, Y., Hara, K., Katoh, R., Tachiya, M. & Furube, A. Femtosecond visible-to-IR spectroscopy of TiO_2_ nanocrystalline films: elucidation of the electron mobility before deep trapping. *J. Phys. Chem. C* **113**, 11741–11746 (2009).

[27] Cooper, J. K. et al. Physical origins of the transient absorption spectra and dynamics in thin-film semiconductors: the case of BiVO_4_. *J. Phys. Chem. C* **122**, 20642–20652 (2018).

[28] Barroso, M., Pendlebury, S. R., Cowan, A. J. & Durrant, J. R. Charge carrier trapping, recombination and transfer in hematite (α-Fe_2_O_3_) water splitting photoanodes. *Chem. Sci.* **4**, 2724 (2013).

[29] Forster, M. et al. Oxygen deficient α-Fe_2_O_3_ photoelectrodes: a balance between enhanced electrical properties and trap-mediated losses. *Chem. Sci.* **6**, 4009–4016 (2015). 28717462 10.1039/c5sc00423c PMC5497273

[30] Selim, S. et al. Impact of oxygen vacancy occupancy on charge carrier dynamics in BiVO_4_ photoanodes. *J. Am. Chem. Soc.* **141**, 18791–18798 (2019). 31663329 10.1021/jacs.9b09056

[31] Fan, Y., Lin, Y., Wang, K., Zhang, K. H. L. & Yang, Y. Intrinsic polaronic photocarrier dynamics in hematite. *Phys. Rev. B* **103**, 085206 (2021).

[32] Godin, R., Wang, Y., Zwijnenburg, M. A., Tang, J. & Durrant, J. R. Time-resolved spectroscopic investigation of charge trapping in carbon nitrides photocatalysts for hydrogen generation. *J. Am. Chem. Soc.* **139**, 5216–5224 (2017). 28319382 10.1021/jacs.7b01547

[33] Norris, D. J., Sacra, A., Murray, C. B. & Bawendi, M. G. Measurement of the size dependent hole spectrum in CdSe quantum dots. *Phys. Rev. Lett.* **72**, 2612–2615 (1994). 10055928 10.1103/PhysRevLett.72.2612

[34] Klimov, V. I. Optical nonlinearities and ultrafast carrier dynamics in semiconductor nanocrystals. *J. Phys. Chem. B* **104**, 6112–6123 (2000).

[35] Scholz, M., Flender, O., Oum, K. & Lenzer, T. Pronounced exciton dynamics in the vacancy-ordered bismuth halide perovskite (CH_3_NH_3_) _3_Bi_2_I_9_ observed by ultrafast UV–vis–NIR transient absorption spectroscopy. *J. Phys. Chem. C* **121**, 12110–12116 (2017).

[36] Sadighian, J. C., Wilson, K. S., Crawford, M. L. & Wong, C. Y. Evolving stark effect during growth of perovskite nanocrystals measured using transient absorption. *Front. Chem.* **8**, 585853 (2020). 33195083 10.3389/fchem.2020.585853 PMC7594514

[37] Bouduban, M. E. F., Burgos-Caminal, A., Ossola, R., Teuscher, J. & Moser, J.-E. Energy and charge transfer cascade in methylammonium lead bromide perovskite nanoparticle aggregates. *Chem. Sci.* **8**, 4371–4380 (2017). 28966782 10.1039/c6sc05211h PMC5580314

[38] Sachs, M., Pastor, E., Kafizas, A. & Durrant, J. R. Evaluation of surface state mediated charge recombination in anatase and rutile TiO_2_. *J. Phys. Chem. Lett.* **7**, 3742–3746 (2016). 27564137 10.1021/acs.jpclett.6b01501 PMC5056403

[39] Caspar, J. V., Kober, E. M., Sullivan, B. P. & Meyer, T. J. Application of the energy gap law to the decay of charge-transfer excited states. *J. Am. Chem. Soc.* **104**, 630–632 (1982).

[40] Chen, B. A., Pang, G. T., Lan, X. Q., He, Z. B. & Chen, R. Strong band filling induced significant excited state absorption in MAPbI_3_ under high pump power. *Mater. Today Phys.* **14**, 100228 (2020).

[41] Chang, Y.-H. et al. Insights from transient absorption spectroscopy into electron dynamics along the Ga-gradient in Cu(In, Ga)Se_2_ solar cells. *Adv. Energy Mater.* **11**, 2003446 (2021).

[42] Sachs, M. et al. Effect of oxygen deficiency on the excited state kinetics of WO_3_ and implications for photocatalysis. *Chem. Sci.* **10**, 5667–5677 (2019). 31293751 10.1039/c9sc00693a PMC6563783

[43] Lettieri, S., Capello, V., Santamaria, L. & Maddalena, P. On quantitative analysis of interband recombination dynamics: theory and application to bulk ZnO. *Appl. Phys. Lett.* **103**, 241910 (2013).

[44] Pastor, E. et al. In situ observation of picosecond polaron self-localisation in α-Fe_2_O_3_ photoelectrochemical cells. *Nat. Commun.* **10**, 3962 (2019). 31481691 10.1038/s41467-019-11767-9 PMC6722133

[45] Gawelda, W. et al. Ultrafast nonadiabatic dynamics of [Fe^II^(bpy)_3_]^2+^ in solution. *J. Am. Chem. Soc.* **129**, 8199–8206 (2007). 17559211 10.1021/ja070454x

[46] Zhang, W. et al. Tracking excited-state charge and spin dynamics in iron coordination complexes. *Nature* **509**, 345–348 (2014). 24805234 10.1038/nature13252 PMC5668134

[47] Grave, D. A. et al. Extraction of mobile charge carrier photogeneration yield spectrum of ultrathin-film metal oxide photoanodes for solar water splitting. *Nat. Mater.* **20**, 833–840 (2021). 33875852 10.1038/s41563-021-00955-y

[48] Carneiro, L. M. et al. Excitation-wavelength-dependent small polaron trapping of photoexcited carriers in α-Fe_2_O_3_. *Nat. Mater.* **16**, 819–825 (2017). 28692042 10.1038/nmat4936

[49] Biswas, S., Husek, J., Londo, S. & Baker, L. R. Highly localized charge transfer excitons in metal oxide semiconductors. *Nano Lett.* **18**, 1228–1233 (2018). 29368513 10.1021/acs.nanolett.7b04818

[50] Biswas, S., Wallentine, S., Bandaranayake, S. & Baker, L. R. Controlling polaron formation at hematite surfaces by molecular functionalization probed by XUV reflection-absorption spectroscopy. *J. Chem. Phys.* **151**, 104701 (2019). 31521099 10.1063/1.5115163

[51] Husek, J., Cirri, A., Biswas, S. & Baker, L. R. Surface electron dynamics in hematite (α-Fe_2_O_3_): correlation between ultrafast surface electron trapping and small polaron formation. *Chem. Sci.* **8**, 8170–8178 (2017). 29619171 10.1039/c7sc02826a PMC5861984

[52] Porter, I. J. et al. Photoexcited small polaron formation in goethite (α-FeOOH) nanorods probed by transient extreme ultraviolet spectroscopy. *J. Phys. Chem. Lett.* **9**, 4120–4124 (2018). 29985006 10.1021/acs.jpclett.8b01525

[53] Pelli Cresi, J. S. et al. Ultrafast formation of small polarons and the optical gap in CeO_2_. *J. Phys. Chem. Lett.* **11**, 5686–5691 (2020). 32580554 10.1021/acs.jpclett.0c01590 PMC8008440

[54] Biswas, S., Husek, J., Londo, S. & Baker, L. R. Ultrafast electron trapping and defect-mediated recombination in NiO probed by femtosecond extreme ultraviolet reflection–absorption spectroscopy. *J. Phys. Chem. Lett.* **9**, 5047–5054 (2018). 30091928 10.1021/acs.jpclett.8b01865

[55] Cowan, A. J., Leng, W., Barnes, P. R. F., Klug, D. R. & Durrant, J. R. Charge carrier separation in nanostructured TiO_2_ photoelectrodes for water splitting. *Phys. Chem. Chem. Phys.* **15**, 8772 (2013). 23632463 10.1039/c3cp50318f

[56] Ma, Y., Pendlebury, S. R., Reynal, A., Le Formal, F. & Durrant, J. R. Dynamics of photogenerated holes in undoped BiVO_4_ photoanodes for solar water oxidation. *Chem. Sci.* **5**, 2964–2973 (2014).

[57] Lohaus, C., Klein, A. & Jaegermann, W. Limitation of Fermi level shifts by polaron defect states in hematite photoelectrodes. *Nat. Commun.* **9**, 4309 (2018). 30333488 10.1038/s41467-018-06838-2 PMC6193028

[58] Pastor, E., et al. Electronic defects in metal oxide photocatalysts. *Nat. Rev. Mater*. 10.1038/s41578-022-00433-0 (2022).

[59] Franchini, C., Reticcioli, M., Setvin, M. & Diebold, U. Polarons in materials. *Nat. Rev. Mater*. 10.1038/s41578-021-00289-w (2021).

[60] Johnson, A. S. et al. All-optical seeding of a light-induced phase transition with correlated disorder. *Nat. Phys.* **20**, 970–975 (2024).

[61] Clarke, T. M. & Durrant, J. R. Charge photogeneration in organic solar cells. *Chem. Rev.* **110**, 6736–6767 (2010). 20063869 10.1021/cr900271s

[62] Kay, A. et al. Wavelength dependent photocurrent of hematite photoanodes: reassessing the hole collection length. *J. Phys. Chem. C* **121**, 28287–28292 (2017).

[63] Maeda, K. & Domen, K. New non-oxide photocatalysts designed for overall water splitting under visible light. *J. Phys. Chem. C* **111**, 7851–7861 (2007).

[64] Inoue, Y. et al. Photocatalytic water splitting by RuO_2_-loaded metal oxides and nitrides with *d*^0^- and *d*^10^-related electronic configurations. *Energy Environ. Sci.* **2**, 364 (2009).

[65] Kudo, A. & Miseki, Y. Heterogeneous photocatalyst materials for water splitting. *Chem. Soc. Rev.* **38**, 253–278 (2009). 19088977 10.1039/b800489g

[66] Greiner, M. T. & Lu, Z.-H. Thin-film metal oxides in organic semiconductor devices: their electronic structures, work functions and interfaces. *npg Asia Mater.* **5**, e55 (2013).

[67] Huda, M. N., Al-Jassim, M. M. & Turner, J. A. Mott insulators: an early selection criterion for materials for photoelectrochemical H_2_ production. *J. Renew. Sustain. Energy* **3**, 053101 (2011).

[68] Wenger, O. S. Photoactive complexes with earth-abundant metals. *J. Am. Chem. Soc.* **140**, 13522–13533 (2018). 30351136 10.1021/jacs.8b08822

[69] McCusker, J. K. Electronic structure in the transition metal block and its implications for light harvesting. *Science* **363**, 484–488 (2019). 30705184 10.1126/science.aav9104

[70] Sinha, N. & Wenger, O. S. Photoactive metal-to-ligand charge transfer excited states in 3*d*^6^ complexes with Cr^0^, Mn^I^, Fe^II^, and Co^III^. *J. Am. Chem. Soc.* **145**, 4903–4920 (2023). 36808978 10.1021/jacs.2c13432 PMC9999427

[71] Kjær, K. S. et al. Luminescence and reactivity of a charge-transfer excited iron complex with nanosecond lifetime. *Science* **363**, 249–253 (2019). 30498167 10.1126/science.aau7160

[72] Sachs, M. *Transient spectroscopic studies of disordered semiconductors for solar-driven fuel synthesis*. PhD thesis, *Imperial College London* https://hdl.handle.net/10044/1/100534 (2020).

[73] Tang, N. J. et al. Nanostructured magnetite (Fe_3_O_4_) thin films prepared by sol–gel method. *J. Magn. Magn. Mater.* **282**, 92–95 (2004).

[74] Jeon, H. S. et al. Simple chemical solution deposition of Co_3_O_4_ thin film electrocatalyst for oxygen evolution reaction. *ACS Appl. Mater. Interfaces* **7**, 24550–24555 (2015). 26489005 10.1021/acsami.5b06189

[75] Selim, S. et al. WO_3_/BiVO_4_: impact of charge separation at the timescale of water oxidation. *Chem. Sci.* **10**, 2643–2652 (2019). 30996980 10.1039/c8sc04679d PMC6419945

[76] Galembeck, A. & Alves, O. L. BiVO_4_ thin film preparation by metalorganic decomposition. *Thin Solid Films* **365**, 90–93 (2000).

[77] Quan, Y., YiO, M. H. N., Li, Y., Myers, R. J. & Kafizas, A. Influence of Bi co-catalyst particle size on the photocatalytic activity of BiOI microflowers in Bi/BiOI junctions—a mechanistic study of charge carrier behaviour. *J. Photochem. Photobiol. Chem.* **443**, 114889 (2023).

[78] A collection of magnetic structures with portable cif-type files. *MAGNDATA* https://www.cryst.ehu.es/magndata/

[79] sumo-kgen documentation. *GitHub* https://smtg-bham.github.io/sumo/sumo-kgen.html

[80] sumo-bandplot documentation. *GitHub* https://smtg-bham.github.io/sumo/sumo-bandplot.html

[81] Sachs, M. et al. Data for article "Metal-centred states control carrier lifetimes in transition metal oxide photocatalysts". *Zenodo*10.5281/zenodo.15198620 (2025). 10.1038/s41557-025-01868-y PMC12411273 40603604

