# Supplementary material for: Metal-centred states control carrier lifetimes in transition metal oxide photocatalysts
Source: Nat Chem. 2025 Jul 2;17(9):1348–55. doi: 10.1038/s41557-025-01868-y (PMC12411273; doi:10.1038/s41557-025-01868-y)
Supplement: Supplementary file 1 — Supplementary Figs. 1–26, Discussion and Tables 1 and 2. [file 41557_2025_1868_MOESM1_ESM.pdf]

nature chemistry

Supplementary information https://doi.org/10.1038/s41557-025-01868-y

Metal-centred states control carrier lifetimes in transition metal oxide photocatalysts

In the format provided by the authors and unedited

Contents

1. Structural characterization 2
2. SEM images 8
3. Steady state absorption 10
4. Transient absorption spectroscopy (TAS) 12
5. References 19

1. Structural characterization

Supplementary Table 1 | X-ray diffraction (XRD) parameter summary for the studied thin films. In line with crystallographic convention, the numbers in brackets represent the error on the last significant digits of each value.

	space group	reference	a	b	c	α	β
**$BiVO_4$ standard**	I 1 1 2/b	1	5.1935(3)	5.0898(3)	11.6972(1)	90	90
**$BiVO_4$ sample**			5.1809(10)	5.0904(7)	11.6571(13)	90	90
**CdO standard**	F m -3 m	2	4.6953(1)	4.6953(1)	4.6953(1)	90	90
**CdO sample**			4.6808(1)	4.6808(1)	4.6808(1)	90	90
**Co_3O_4 standard**	F m -3 m	3	8.0720(30)	8.0720(30)	8.0720(30)	90	90
**Co_3O_4 sample**			8.0588(3)	8.0588(3)	8.0588(3)	90	90
**Cr_2O_3 standard**	R -3 c H	4	4.9572(1)	4.9572(1)	13.5917(10)	90	90
**Cr_2O_3 sample**			4.9316(2)	4.9316(2)	13.5093(8)	90	90
**Fe_2O_3 standard**	R -3 c H	5	5.0353(5)	5.0353(5)	13.7495(5)	90	90
**Fe_2O_3 sample**			5.01666(10)	5.01666(10)	13.6741(29)	90	90
**NiO standard**	F m -3 m	6	4.1718(9)	4.1718(9)	4.1718(9)	90	90
**NiO sample**			4.1664(9)	4.1664(9)	4.1664(9)	90	90

	γ	V	V change (%)	τ (nm)	wRp	X^2
**$BiVO_4$ standard**	90.387(4)	309.20(3)	-	-	-	-
**$BiVO_4$ sample**	90.342(14)	307.43(5)	-0.57	29.96	0.0133	1.82
**CdO standard**	90	103.51(1)	-	-	-	-
**CdO sample**	90	102.56(1)	-0.92	47.89	0.0216	3.48
**Co_3O_4 standard**	90	525.95(34)	-	-	-	-
**Co_3O_4 sample**	90	523.37(6)	-0.49	38.55	0.0187	1.35
**Cr_2O_3 standard**	120	289.25(1)	-	-	-	-
**Cr_2O_3 sample**	120	284.54(2)	-1.63	22.72	0.0278	3.07
**Fe_2O_3 standard**	120	301.90(4)	-	-	-	-
**Fe_2O_3 sample**	120	298.03(8)	-1.28	25.42	0.018	1.22
**NiO standard**	90	72.61(3)	-	-	-	-
**NiO sample**	90	72.32(4)	-0.39	7.15	0.0088	1.51

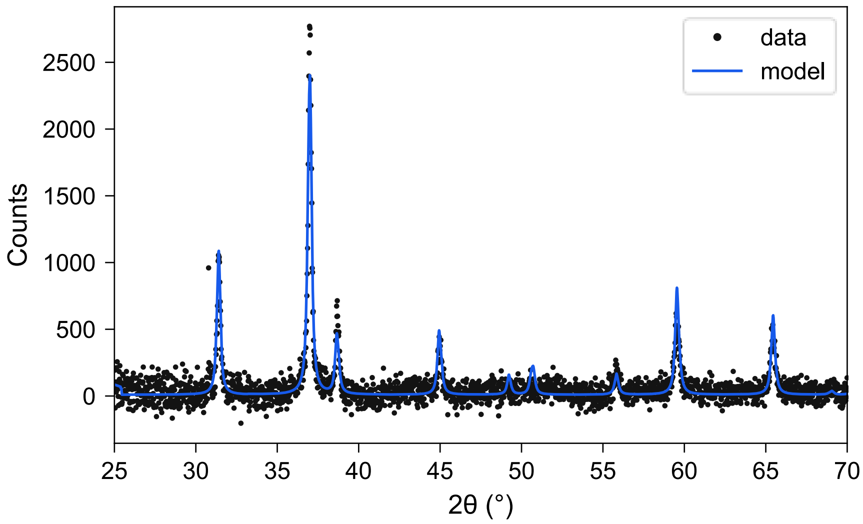

Supplementary Fig. 1 | XRD pattern of our Co_3O_4 thin films, compared to reference data retrieved from the PSDS database as reported in Ref. 3.

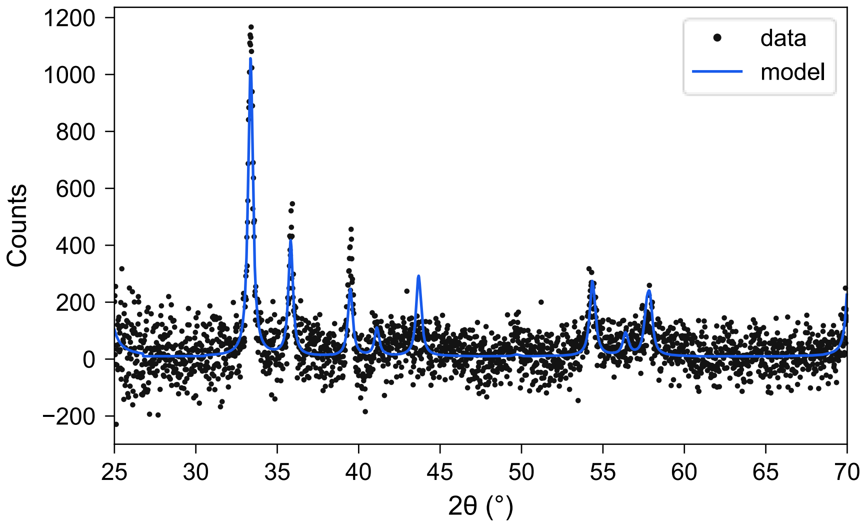

Supplementary Fig. 2 | XRD pattern of our Fe_2O_3 thin films compared to reference data retrieved from the PSDS database as reported in Ref. 5.

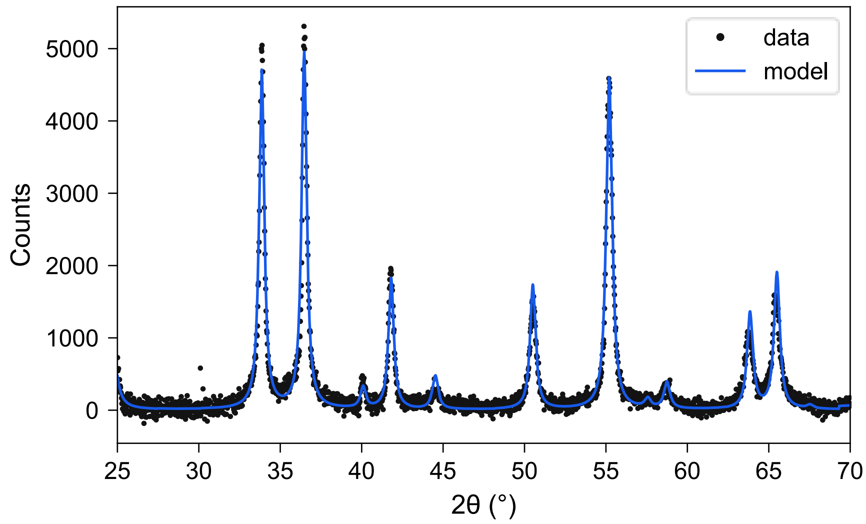

Supplementary Fig. 3 | XRD pattern of our Cr_2O_3 thin films compared to reference data retrieved from the PSDS database as reported in Ref. 4.

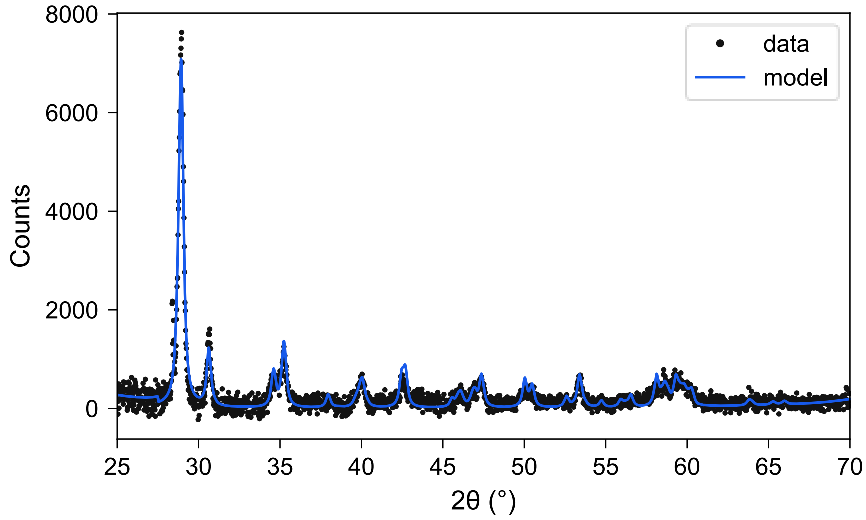

Supplementary Fig. 4 | XRD pattern of our $BiVO_4$ thin films compared reference data retrieved from the PSDS database as reported in Ref. 1.

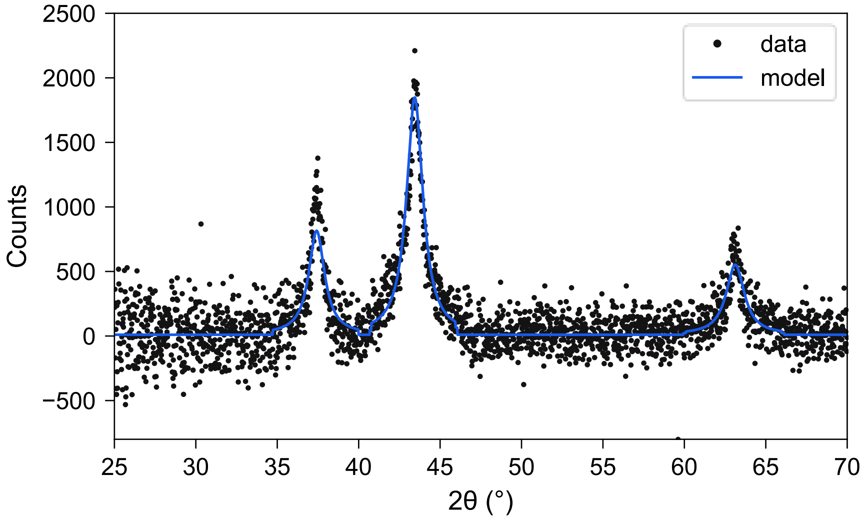

Supplementary Fig. 5 | XRD pattern of our NiO thin films compared to reference data retrieved from the PSDS database as reported in Ref. 6.

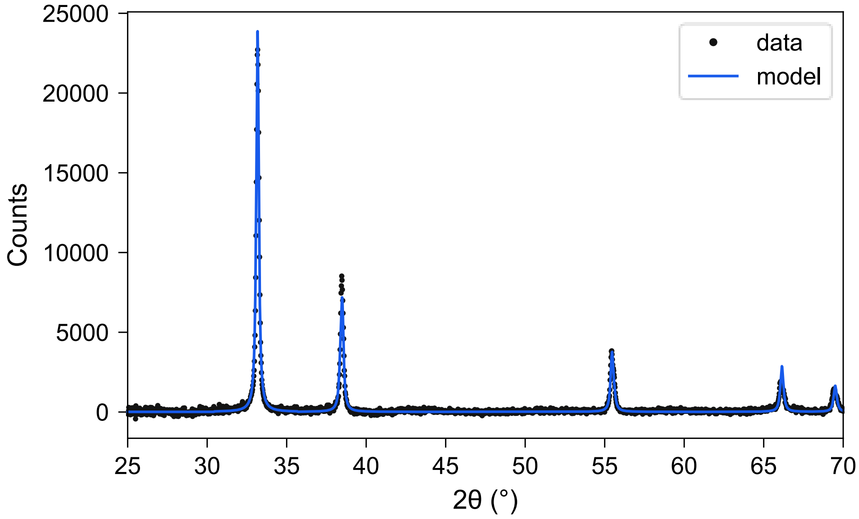

Supplementary Fig. 6 | XRD pattern of our CdO thin films compared to reference data retrieved from the PSDS database as reported in Ref. 2.

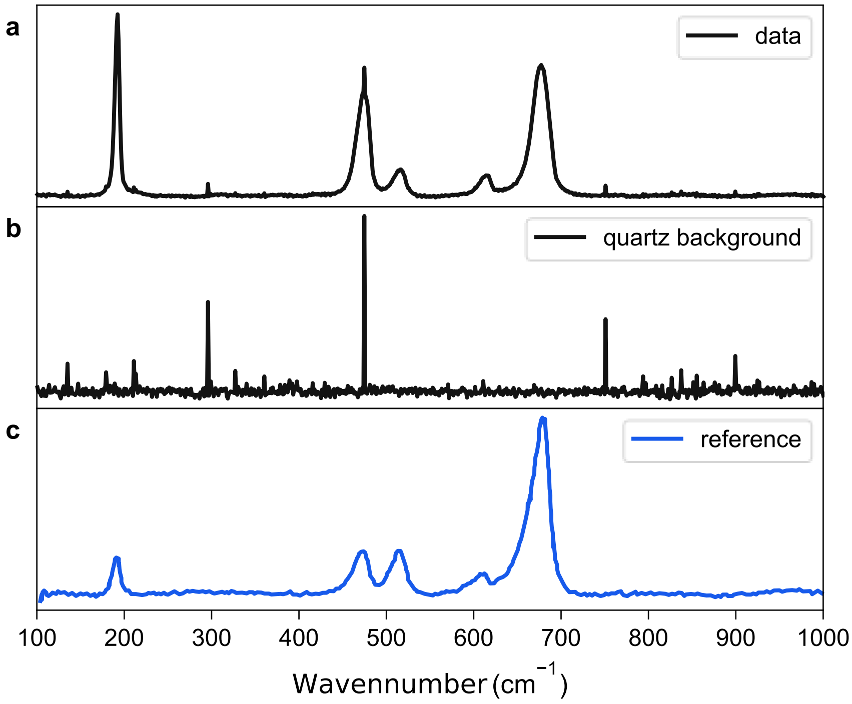

Supplementary Fig. 7 | Raman spectra of (**a**) our Co_3O_4 thin films recorded using a 633 nm laser, (**b**) the instrument background, recorded using a blank quartz substrate, and (**c**) Co_3O_4 reference data recorded using a 532 nm laser, digitized and replotted here from Ref. 7.

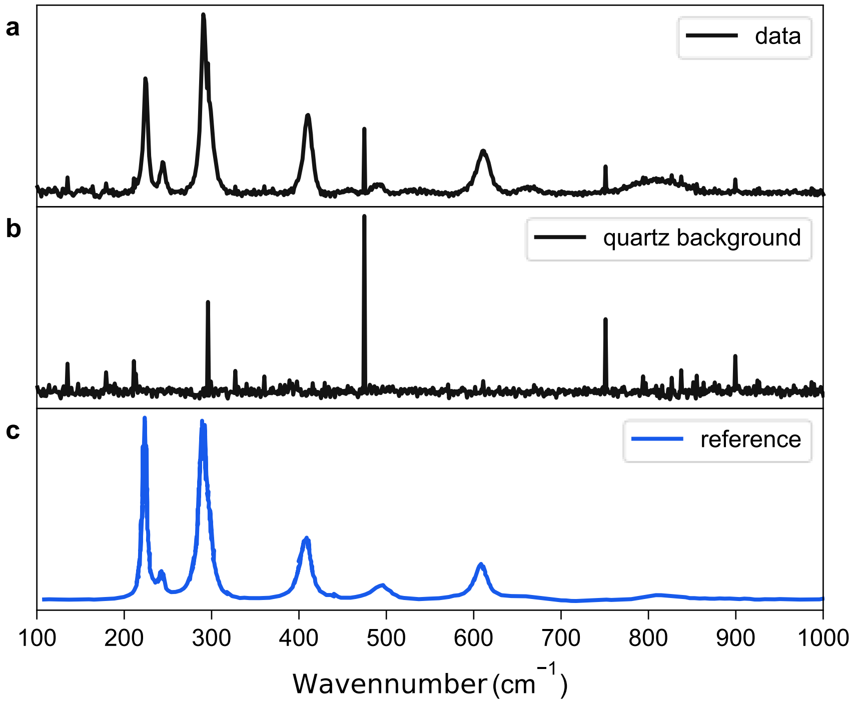

Supplementary Fig. 8 | Raman spectra of (**a**) our Fe_2O_3 thin films recorded using a 633 nm laser, (**b**) the instrument background, recorded using a blank quartz substrate, and (**c**) Fe_2O_3 reference data recorded using a 633 nm laser, digitized and replotted here from Ref. 8.

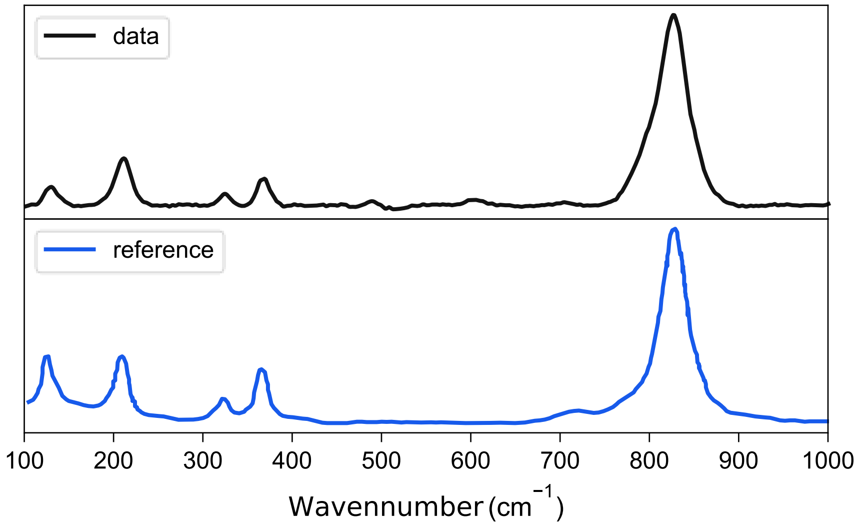

Supplementary Fig. 9 | Raman spectra of our $BiVO_4$ thin films (top) recorded using a 532 nm laser, compared to $BiVO_4$ reference data digitized and replotted here from Ref. 9 (bottom), recorded using a 530 nm laser.

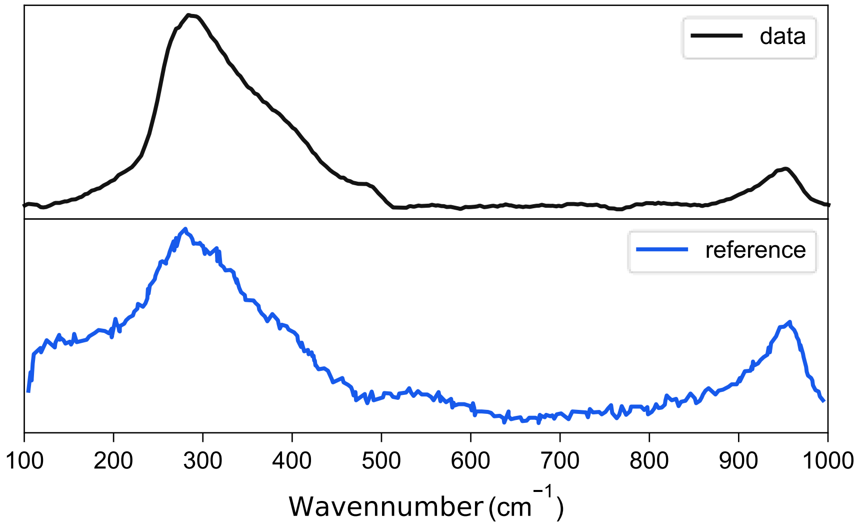

Supplementary Fig. 10 | Raman spectrum of our CdO thin films (top) compared to CdO reference data, digitized and replotted here from Ref. 10 (bottom). Both datasets were recorded using a 532 nm laser.

2. SEM images

To calculate the volumetric charge carrier densities shown in **Fig. 3a-d** in the main text, we estimate the thicknesses of the relevant films from cross-section SEM images.

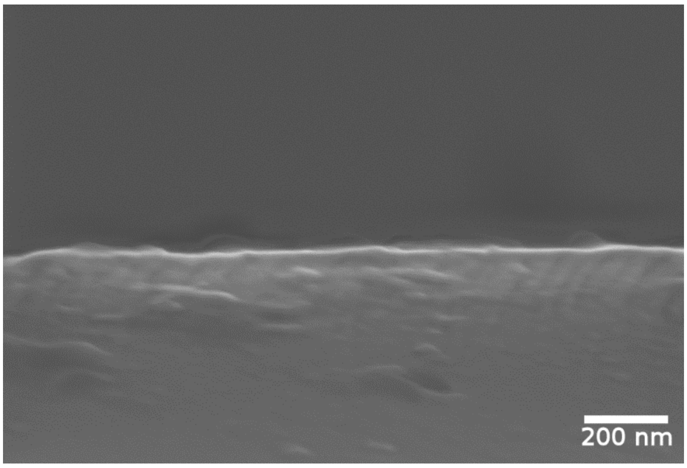

Supplementary Fig. 11 | Cross section SEM image of one of our Cr_2O_3 thin films, with an estimated average film thickness of 30 nm.

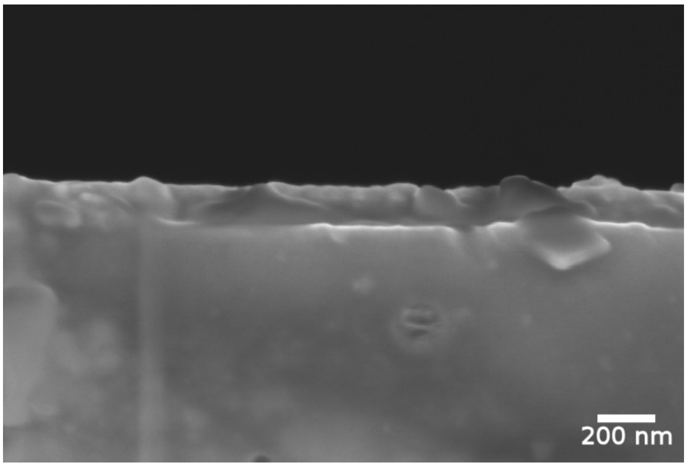

Supplementary Fig. 12 | Cross section SEM image of one of our Fe_2O_3 thin films, with an estimated average film thickness of 130 nm.

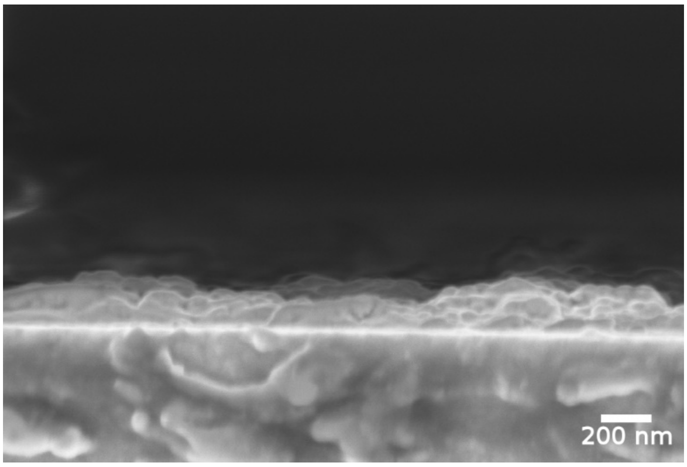

Supplementary Fig. 13 | Cross section SEM image of one of our Co_3O_4 thin films, with an estimated average film thickness of 70 nm.

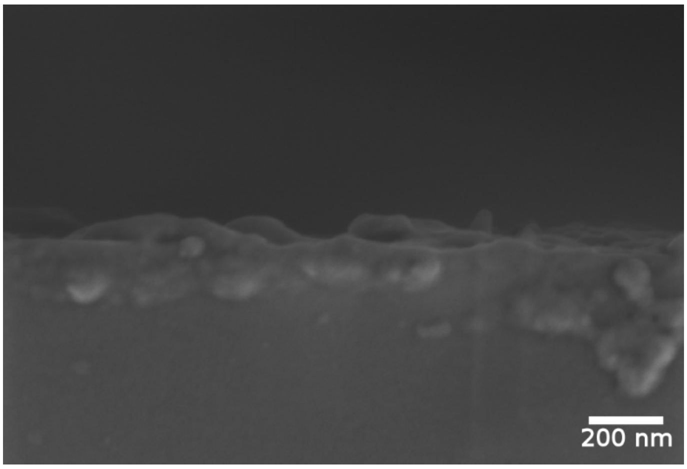

Supplementary Fig. 14 | Cross section SEM image of one of our $BiVO_4$ thin films, with an estimated average film thickness of 100 nm.

3. Steady state absorption

Direct bandgap energies, indicated by dashed red lines in **Fig. 1** in the main text, were determined via Tauc plots, yielding values of 1.51 eV for Co_3O_4, 3.16 eV for Cr_2O_3, and 2.2 eV for Fe_2O_3, in good agreement with literature reports.[11–16]

The temperature dependence of the optical gap can be modelled using the following relation proposed by O'Donnell and Chen,[17] based on the early work of Manoogian and Leclerc[18,19]:

$$\mathrm{E_g}(T) = E_g(0) - S * E_{ph} * \left(\coth\left(\frac{E_{ph}}{2k_BT}\right) - 1\right) \qquad \text{(Eq. 1)}$$

where E_g (0) is the bandgap at 0 K, S is a dimensionless constant which characterizes the electron-phonon coupling strength, and E_{ph} is an average phonon energy. The full red lines in **Supplementary Fig. 15b**, **Supplementary Fig. 16b**, and **Supplementary Fig. 17b** show the fits to the data and the extracted parameters are summarised in **Supplementary Table 2.** Fe_2O_3 exhibits a substantially smaller S than Cr_2O_3 or Co_3O_4, which suggests lower electron-phonon coupling. This reduced electron-phonon coupling in Fe_2O_3 is in line with its more pronounced bimolecular behaviour as the interaction with the lattice required for monomolecular polaron formation is reduced. We also note that Fe_2O_3 has the smallest E_{ph} of 28 meV, which matches its lowest energy Raman active transition at 225 cm^{-1} (**Supplementary Fig. 8**).

Supplementary Table 2 | Fit parameters obtained from **Eq. 1**: bandgap energy E_g at zero Kelvin, coupling constant S, average phonon energy E_{ph}.

	E_g(0 K) (eV)	S	E_{ph} (meV)
**Fe_2O_3**	2.20	0.59	28
**Cr_2O_3**	3.16	1.02	34
**Co_3O_4**	1.51	0.90	43

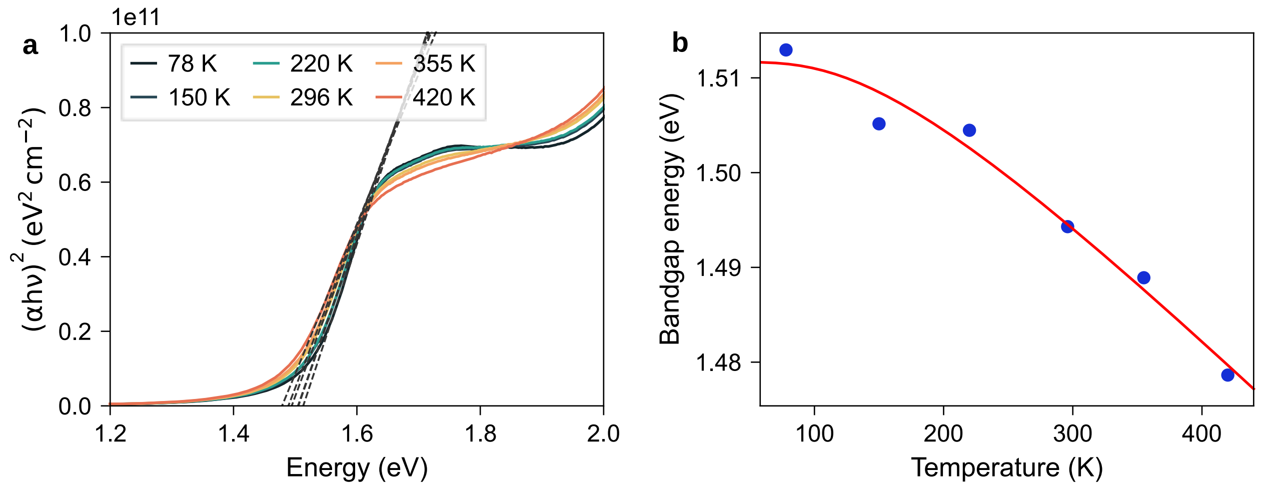

Supplementary Fig. 15 | Temperature dependent absorption onset for Co_3O_4. (**a**) Direct bandgap Tauc plot and (**b**) fit of the bandgap as a function of temperature, extrapolating to 1.51 eV at 0K.

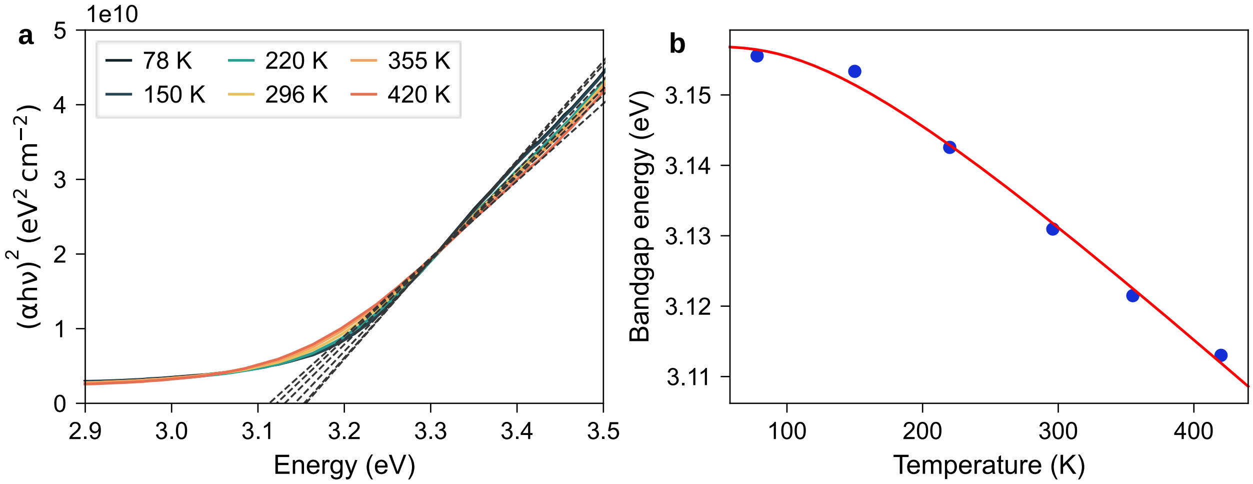

Supplementary Fig. 16 | Temperature dependent absorption onset for Cr_2O_3. (**a**) Direct bandgap Tauc plot and (**b**) fit of the bandgap as a function of temperature, extrapolating to 3.16 eV at 0K.

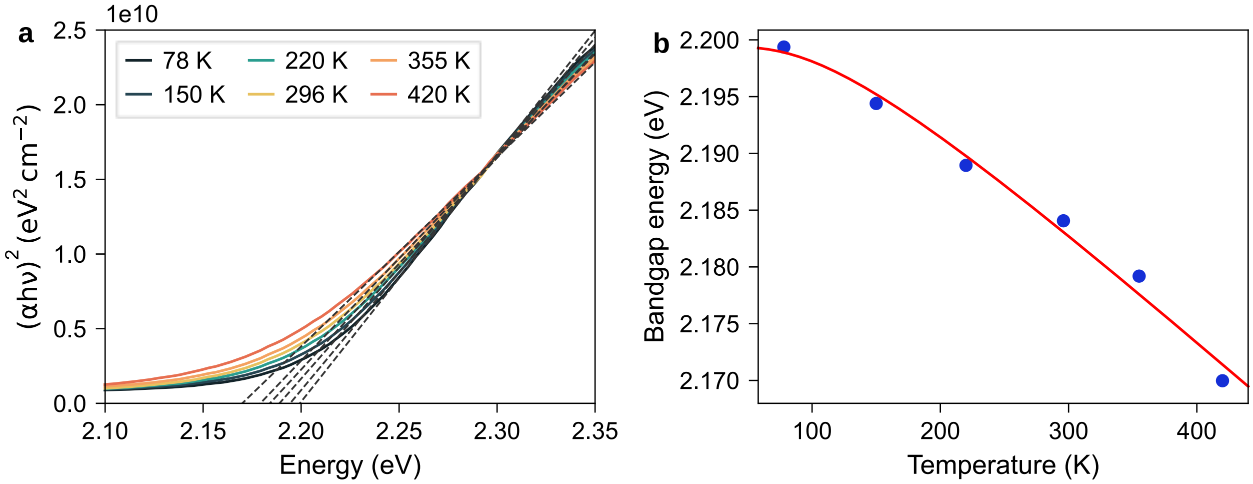

Supplementary Fig. 17 | Temperature dependent absorption onset for Fe_2O_3. (**a**) Direct bandgap Tauc plot and (**b**) fit of the bandgap as a function of temperature, extrapolating to 2.2 eV at 0K.

4. Transient absorption spectroscopy (TAS)

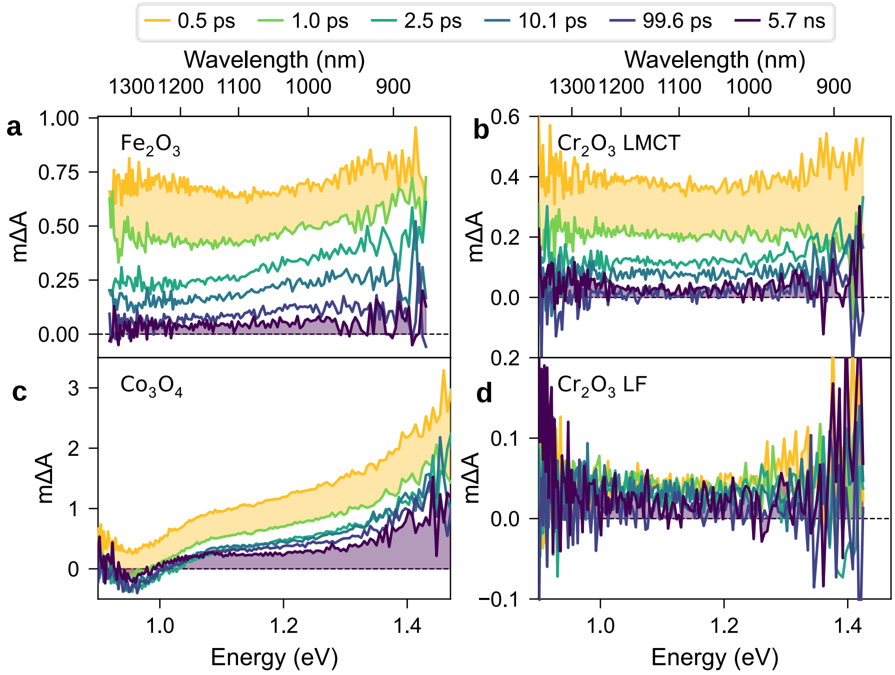

Supplementary Fig. 18 | Transient absorption spectra for (**a**) Fe_2O_3, (**b**) Co_3O_4, (**c**) Cr_2O_3 upon LMCT excitation (3.10 eV, 1.68 eV, and 3.40 eV, respectively). (**d**) Transient absorption spectra for Cr_2O_3 upon LF excitation (2.7 eV). The filled yellow and purple regions illustrate the shape of the broad and structured components discussed in the main text, originating from direct carrier absorption and the Stark effect, respectively. Note that the same number of photons absorbed were used for comparing LF and LMCT in Cr_2O_3. The data shown here is identical to the NIR data shown in Figure 2 but leaves out the visible part to facilitate an evaluation of spectral changes in the NIR range.

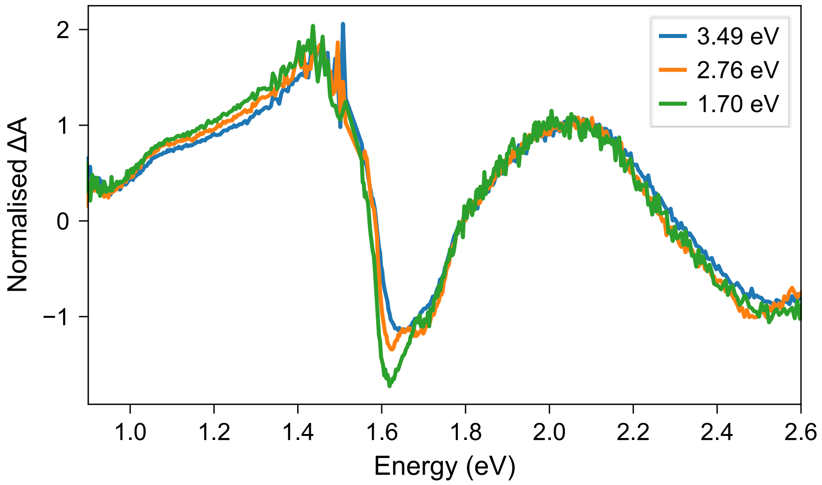

Supplementary Fig. 19 | Transient spectra of Co_3O_4 for different excitation energies, probed at 0.3 ps. The observation of near identical spectral shape suggests that the same species are generated in all cases. Laser fluences were adjusted to yield ~2e19 absorbed photons per cm^3 for all excitation energies.

Assignment of the structured component to trapped charges

There are three typically types of possible perturbations for each of the individual optical transitions that make up the overall steady state absorbance shown in **Fig. 1** in the main text: an absorption peak may change in amplitude due to a change in transition strength, shift in energy, or change its width. A change in transition strength leaves the general shape of the peak unchanged, a peak shift will result in a shape which matches the first derivative of the original peak, and peak broadening will yield a second-derivative-like shape. A comparison of the transient spectra at 5.7 ns reveals an appreciable agreement with the second derivative of their steady state absorbance spectra as shown in **Supplementary Fig. 21**, suggesting that the structured component primarily arises from a broadening of the underlying optical transitions upon photoexcitation. A certain degree of mismatch can be expected, as the second derivative of the entire absorbance spectrum implies that all transitions broaden to the same extent, which is unlikely to be the case in practice.

One possible reason for such a second derivative like transient spectrum is a Stark effect, meaning that the electric field between photogenerated charges perturbs the optical absorption of the excited semiconductor. A second-derivative-like Stark effect is indicative of a change in dipole moment between the excited state and the ground state, consistent with the presence of trapped charges which act like permanent dipoles on the timescale of their own lifetime. Experiments at microsecond and longer delay times show that the structured signal persists until much longer timescales and decays with power law dynamics (**Supplementary Fig. 20**), which further supports an assignment to trapped charges.

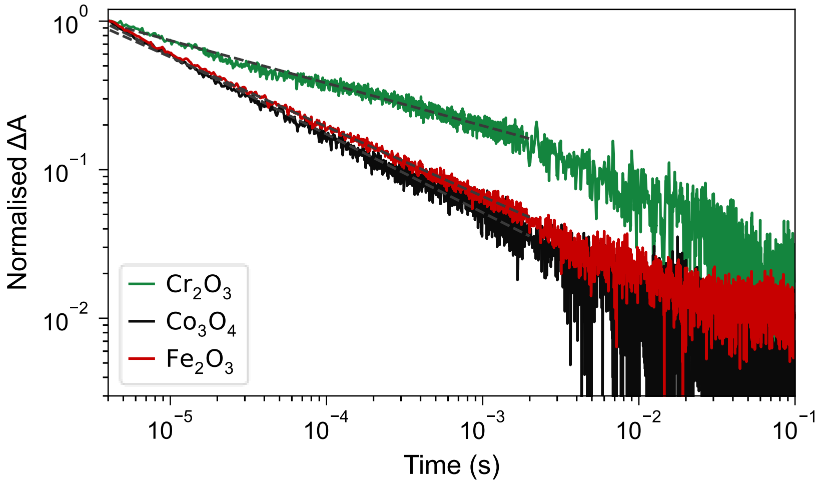

Supplementary Fig. 20 | Transient kinetics probed for the structured component (see main text) following 355 nm excitation (2.0 mJ/cm^2 for Cr_2O_3 and 0.8 mJ/cm^2 for Co_3O_4 and Fe_2O_3). The kinetics were probed near the absorption maxima of the structured component: 1.91 eV (650 nm) for Cr_2O_3, and 2.25 eV (550 nm) for Co_3O_4 and Fe_2O_3. Slopes were found to be independent of the used laser fluence. The dashed lines represent linear fits to the log-log transformed data (slopes: -0.29 for Cr_2O_3, -0.53 for Co_3O_4, -0.47 for Fe_2O_3), suggesting that the decay of the structured component follows a power law decay, consistent with trap-mediated charge recombination. Different slopes are indicative of different trap state energies that control the mobility and recombination dynamics of these charge carriers.[20]

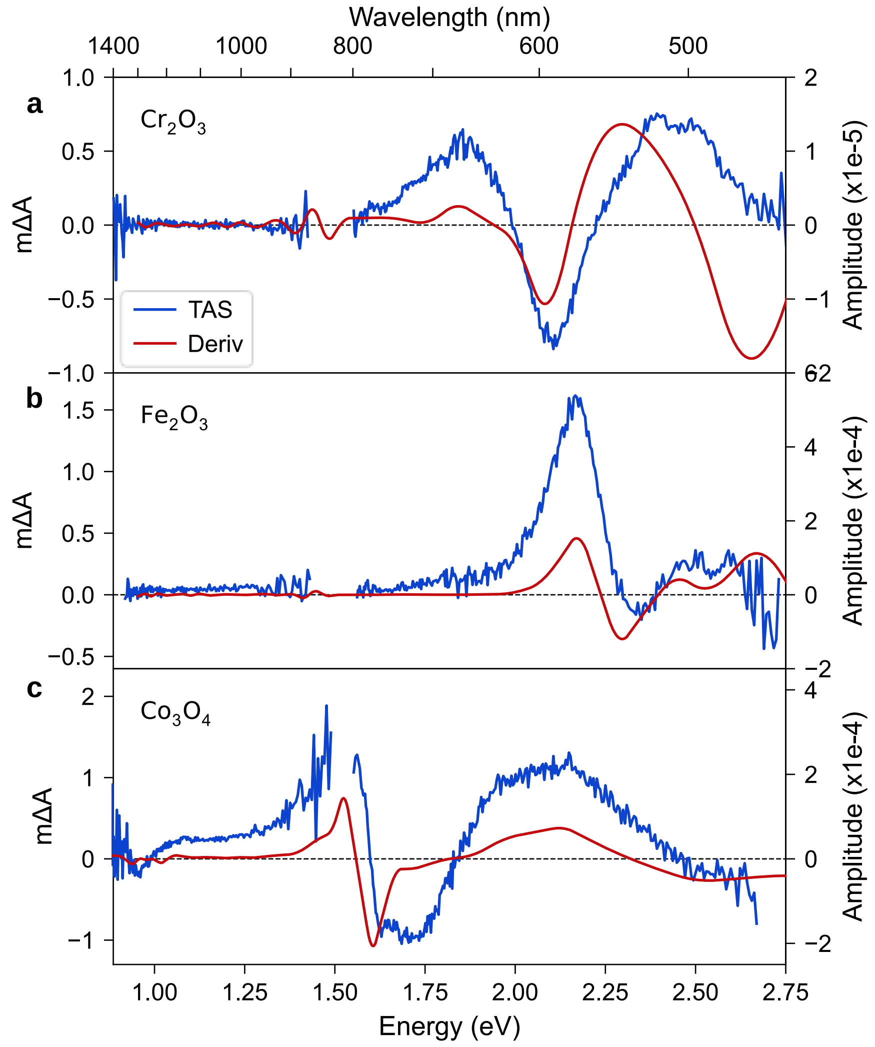

Supplementary Fig. 21 | Comparison of transient absorption spectrum, probed at 5.7 ns (blue traces), and the second derivative of the steady state absorbance spectrum (red traces) for **(a)** Cr_2O_3, **(b)** Fe_2O_3, and **(c)** Co_3O_4.

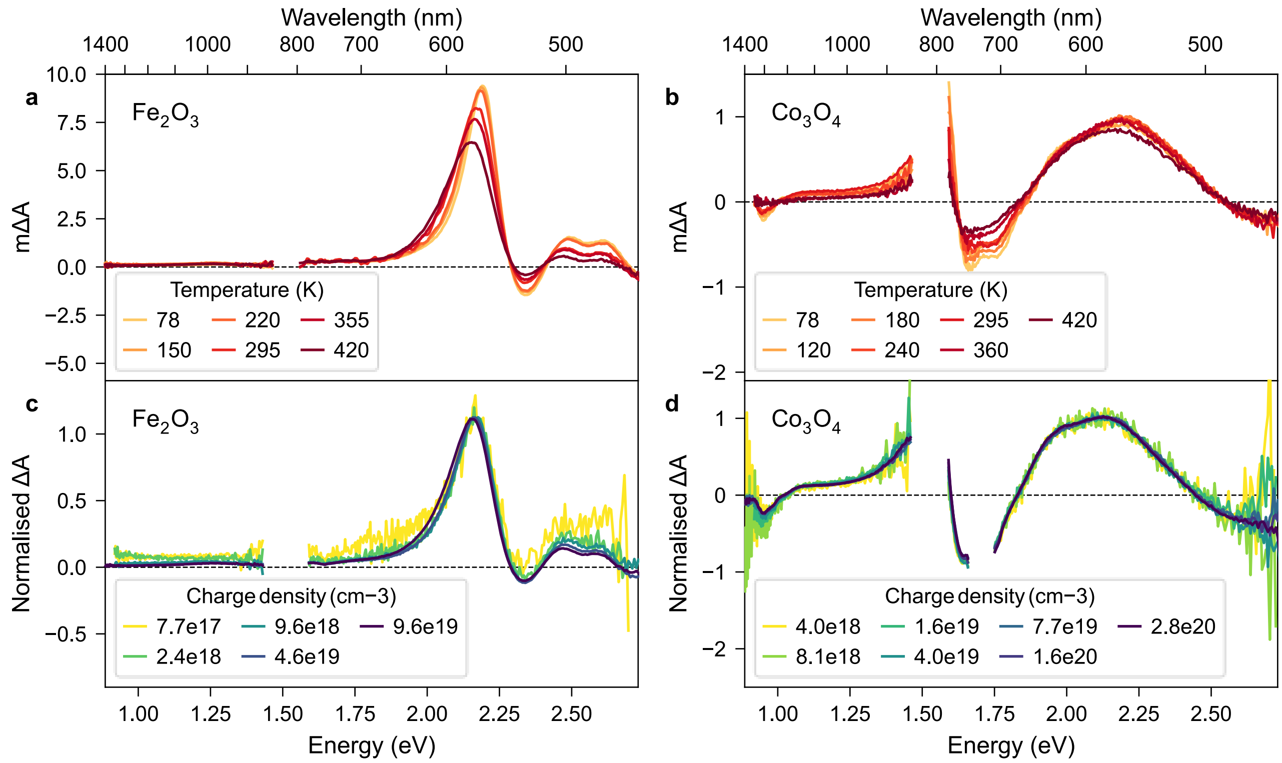

Supplementary Fig. 22 | Transient absorption spectra for Fe_2O_3 and Co_3O_4, averaged over a 1 - 6 ns range, showing the structured component recorded **(a-b)** as a function of temperature at a fluence corresponding to ~2e19 absorbed photons per cm^3, and **(c-d)** as a function of laser fluence at 295 K. The spectral shape predominantly changes as a function of temperature, demonstrating that the signal is controlled by the environmental temperature rather than laser heating (if laser heating was the controlling factor, the spectral shape would be expected to change predominantly with laser fluence).

Assignment of transient features to photogenerated charges

In the following section we justify the assignment of our transient absorption features to photogenerated charges, as opposed to an assignment to laser heating in some recent studies.[21–23]

When recording transient spectra at increasingly higher ambient temperature we observe spectral shifts towards lower energy (**Supplementary Fig. 22a-b**), which we associate with temperature-induced bandgap shrinking, even at the higher fluences used for the measurement of ~1 mJ cm^{-2}. This shift suggests that the transient absorption response is sensitive to the ambient temperature, and any transient heating effects persisting on the ns timescale should therefore be much smaller than the temperature steps of ~70 K. In contrast, the laser fluence, and thus the photogenerated carrier density, has a negligible effect on the spectral shape (**Supplementary Fig. 22c-d**).

Deeply trapped charges, to which we assign the structured spectral features shown in **Supplementary Fig. 22**, can be monitored via their associated Stark effect signals and eventually recombine via trap-limited recombination on timescales up to milliseconds.[24] The recombination of a majority carrier polaron and a deeply trapped minority charge is often non-radiative, although it can be radiative in some systems such as ZnO.[25,26] Aside from trapping upon photoexcitation, trapped minority charges can also be generated via thermal excitation of majority carriers out of these trap states, which has caused considerable debate on whether these long-lived transient features are caused by laser heating.[21–23] Our measurements as a function of excitation fluence and temperature

suggest that transient spectra are primarily sensitive to the ambient temperature rather than the used laser fluence (**Supplementary Fig. 22**). Spectral features associated with trapped charges have been found to be absent in monocrystalline Fe_2O_3 films[27] and present in polycrystalline TMO films,[24,28] suggesting a correlation to defect populations, consistent with our previous analysis of this point for $BiVO_4$.[24]

Apart from photoexcitation, the structured signal can also be induced via other stimuli such as an increase in temperature, as pointed out in recent studies.[21,29] We consider that an increase in temperature or applied potential, as well as direct defect excitation, generates minority carriers in sub-bandgap states, just like the minority carrier trapping from a band state.

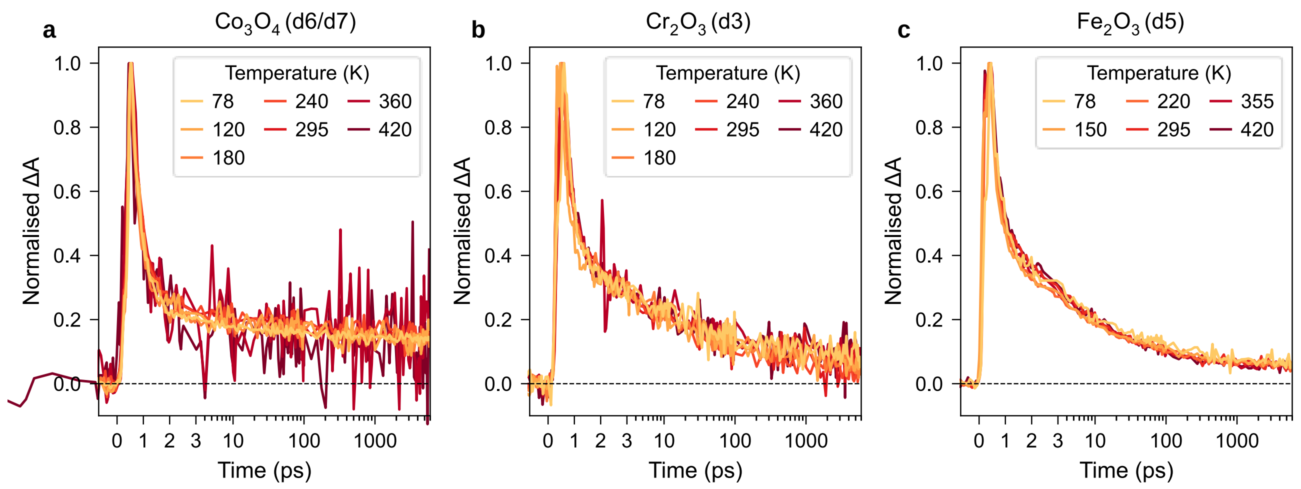

Supplementary Fig. 23 | Normalised transient absorption kinetics for **(a)** Co_3O_4, **(b)** Cr_2O_3, and **(c)** Fe_2O_3 as a function of temperature, recorded at a fluence corresponding to ~2e19 absorbed photons per cm^3 and probed at 1.13 eV (1100 nm). The d-orbital occupancy is indicated in brackets on top of each plot. The kinetics have been normalised to compensate for the changes in amplitude shown in **Supplementary Fig. 22a-b** to facilitate a comparison of the kinetic profile.

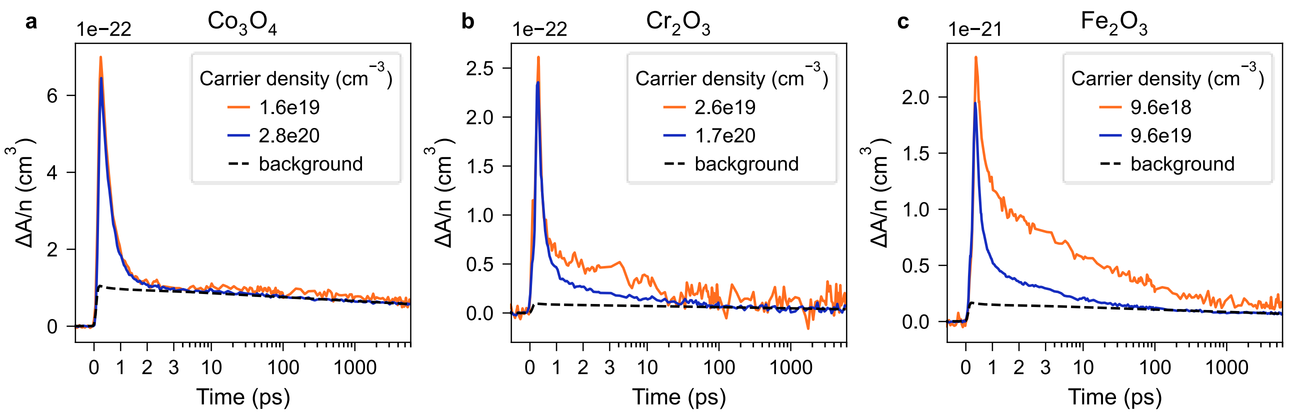

Supplementary Fig. 24 | Background subtraction for the transient absorption kinetics shown in **Fig. 3e** in the main text for **(a)** Co_3O_4, **(b)** Cr_2O_3, and **(c)** Fe_2O_3. As is clearest for Co_3O_4, the background from the structured component decays linearly on a log-lin plot. The background is therefore approximated through a linear fit of the high fluence transient data (where the contribution from any

biomolecular component is minimised) above 100 ps, carried out on a log-lin plot. This background is then convolved with a gaussian instrument response and subtracted from the lower fluence data.

Transient response of NiO

NiO metal oxide has a d^8 configuration and its transient response follows the trend described for open d-shell oxides: a rapidly decaying broad transient absorption feature is observed at energies smaller than the bandgap, i.e. over the entire probe range shown in **Supplementary Fig. 25b**, which we attribute to the depletion of a band-like state through LF relaxation (shaded in yellow like in **Fig. 2** in the main text). The transient kinetics shown in **Supplementary Fig. 25c** further illustrate the presence of the carrier density independent LF relaxation process on the sub-ps timescale. Like Cr_2O_3 and Fe_2O_3, NiO also exhibits a carrier density dependent recombination process at longer time, assigned to the slower bimolecular recombination of polaronic charges.

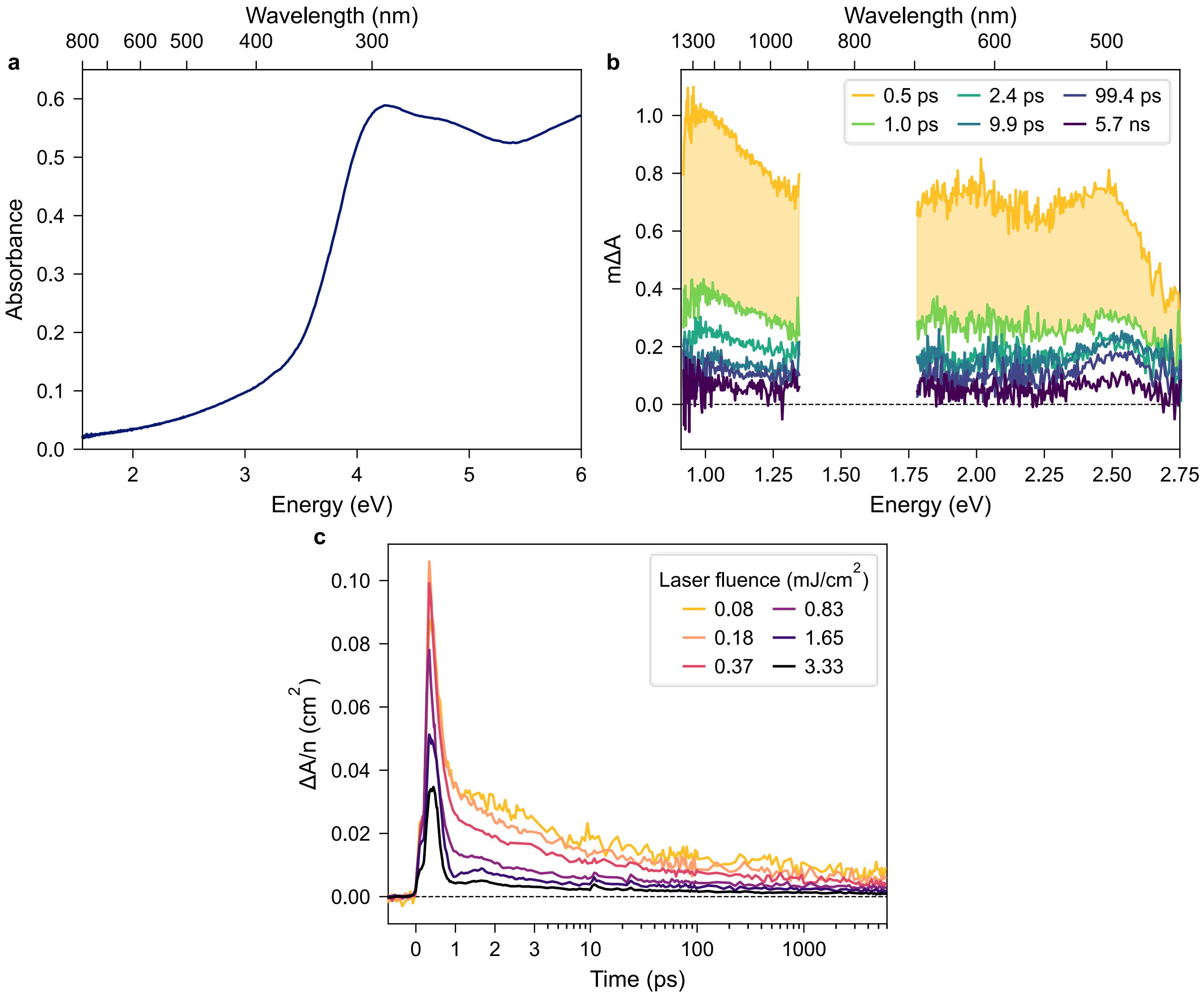

Supplementary Fig. 25 | Optical data for a polycrystalline NiO film. **(a)** Steady state absorbance spectrum. **(b)** Transient absorption spectra as a function of time, recorded using a laser fluence of 0.37 mJ cm^{-2}. **(c)** Transient absorption kinetics as a function of laser fluence, probed at 1.13 eV (1100 nm) and normalised via division by the used laser fluence. All transient data was recorded upon excitation at 4.13 eV (300 nm).

Transient response of CdO

CdO is a d[10] TMO and exhibits photophysical signatures which are distinctly different from oxides with d[0] or open d-shell configurations. While reactive charges appear as a broad excited state absorption feature at sub-bandgap energies in TMOs with empty or open d-shells, they manifest in the form of a near-band edge bleach in closed shell TMOs. Regardless of spectral signatures, we find that oxides with d[10] configuration are similar to d[0] oxides in the sense that they are able to generate long-lived charges due to the absence of LF relaxation. As mentioned, the main transient feature representing these long-lived charges is a bleach near the band edge (**Supplementary Fig. 26b**). A shift of this bleach towards lower energy can be observed on the sub-ps timescale, consistent with an energy loss of photogenerated charges as they undergo polaronic relaxation. The decay of these polaronic charges is strongly fluence dependent (**Supplementary Fig. 26c**): at low fluence, photogenerated charges undergo pseudo-first order recombination with intrinsic carriers, whereas bimolecular recombination of photogenerated charges becomes dominant at higher fluences due to the photogenerated carrier density outweighing the intrinsic carrier density.

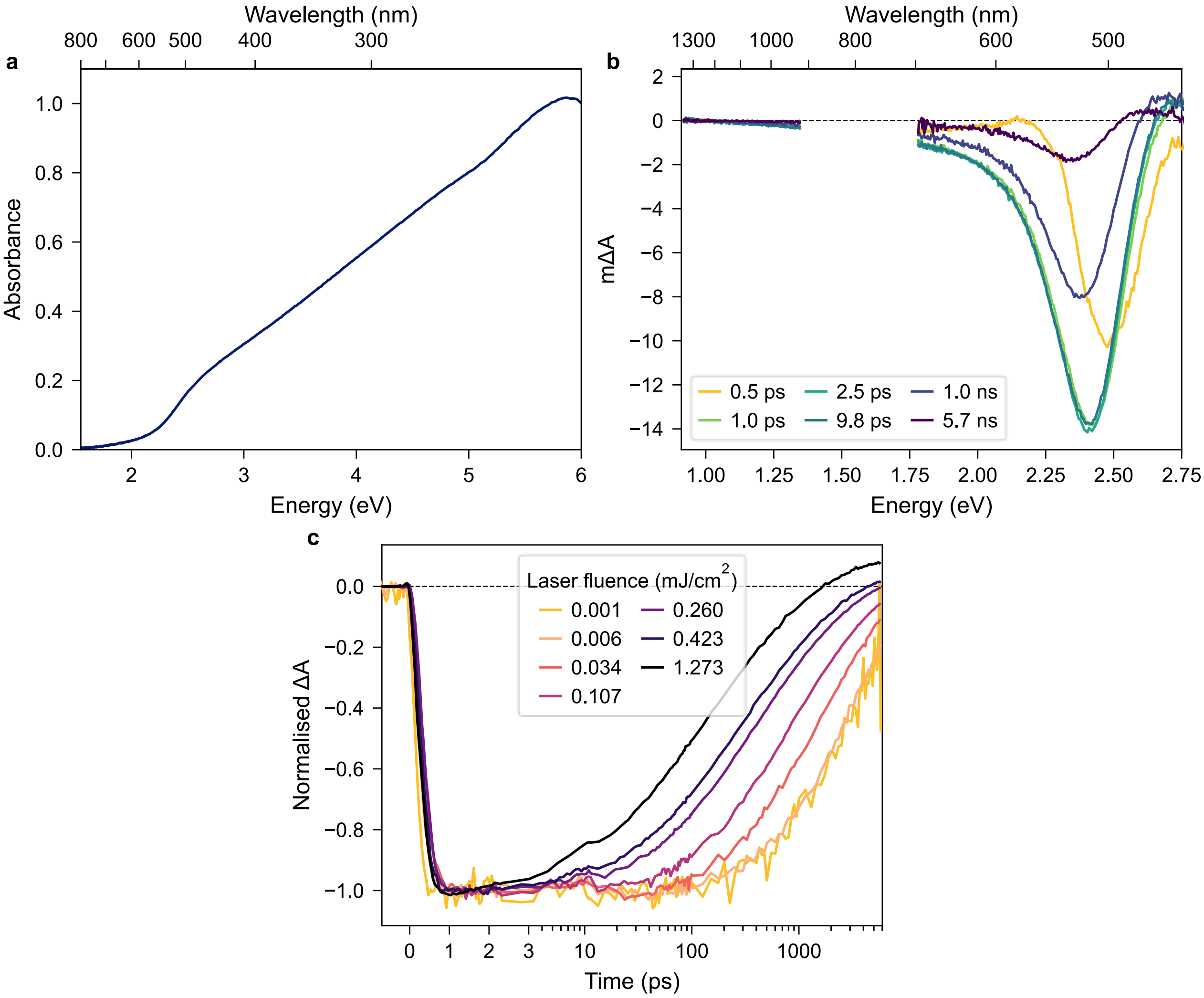

Supplementary Fig. 26 | Optical data for a polycrystalline CdO film. **(a)** Steady state absorbance spectrum. **(b)** Transient absorption spectra as a function of time, recorded using a laser fluence of 0.034 mJ cm^{-2}. **(c)** Normalised transient absorption kinetics as a function of laser fluence, probed at 2.40 eV (516 nm). All transient data was recorded upon excitation at 3.54 eV (350 nm).

5. References

(1) Sleight, A. W.; Chen, H. -y.; Ferretti, A.; Cox, D. E. Crystal Growth and Structure of BiVO4. *Mater. Res. Bull.* **1979**, *14* (12), 1571–1581. https://doi.org/10.1016/0025-5408(72)90227-9.

(2) Taylor, D. Thermal Expansion Data. I: Binary Oxides with the Sodium Chloride and Wurtzite Structures, MO. *Therm. Expans. Data Bin. Oxides Sodium Chloride Wurtzite Struct. MO* **1984**, *83* (1), 5–9.

(3) Picard, J. P.; Baud, G.; Besse, J. P.; Chevalier, R. Croissance Cristalline et Étude Structurale de Co3O4. *J. Common Met.* **1980**, *75* (1), 99–104. https://doi.org/10.1016/0022-5088(80)90373-2.

(4) Hill, A. H.; Harrison, A.; Dickinson, C.; Zhou, W.; Kockelmann, W. Crystallographic and Magnetic Studies of Mesoporous Eskolaite, Cr2O3. *Microporous Mesoporous Mater.* **2010**, *130* (1), 280–286. https://doi.org/10.1016/j.micromeso.2009.11.021.

(5) Sawada, H. An Electron Density Residual Study of α-Ferric Oxide. *Mater. Res. Bull.* **1996**, *31* (2), 141–146. https://doi.org/10.1016/0025-5408(95)00183-2.

(6) Singh, J.; Lee, S.; Kim, S.; Singh, S. P.; Kim, J.; Rai, A. K. Fabrication of 1D Mesoporous NiO Nano-Rods as High Capacity and Long-Life Anode Material for Lithium Ion Batteries. *J. Alloys Compd.* **2021**, *850*, 156755. https://doi.org/10.1016/j.jallcom.2020.156755.

(7) Deng, S.; Chen, N.; Deng, D.; Li, Y.; Xing, X.; Wang, Y. Meso- and Macroporous Coral-like Co3O4 for VOCs Gas Sensor. *Ceram. Int.* **2015**, *41* (9, Part A), 11004–11012. https://doi.org/10.1016/j.ceramint.2015.05.045.

(8) Lassoued, A.; Lassoued, M. S.; Dkhil, B.; Ammar, S.; Gadri, A. Synthesis, Photoluminescence and Magnetic Properties of Iron Oxide (α-Fe2O3) Nanoparticles through Precipitation or Hydrothermal Methods. *Phys. E Low-Dimens. Syst. Nanostructures* **2018**, *101*, 212–219. https://doi.org/10.1016/j.physe.2018.04.009.

(9) Gu, S.; Li, W.; Wang, F.; Li, H.; Zhou, H. Substitution of Ce(III,IV) Ions for Bi in BiVO4 and Its Enhanced Impact on Visible Light-Driven Photocatalytic Activities. *Catal. Sci. Technol.* **2016**, *6* (6), 1870–1881. https://doi.org/10.1039/C5CY01412C.

(10) Kokane, S. B.; Sartale, S. D.; Girija, K. G.; Jagannath; Sasikala, R. Photocatalytic Performance of Pd Decorated TiO2–CdO Composite: Role of in Situ Formed CdS in the Photocatalytic Activity. *Int. J. Hydrog. Energy* **2015**, *40* (39), 13431–13442. https://doi.org/10.1016/j.ijhydene.2015.08.037.

(11) Jeon, T. H.; Moon, G.; Park, H.; Choi, W. Ultra-Efficient and Durable Photoelectrochemical Water Oxidation Using Elaborately Designed Hematite Nanorod Arrays. *Nano Energy* **2017**, *39*, 211–218. https://doi.org/10.1016/j.nanoen.2017.06.049.

(12) Kim, J. Y.; Magesh, G.; Youn, D. H.; Jang, J.-W.; Kubota, J.; Domen, K.; Lee, J. S. Single-Crystalline, Wormlike Hematite Photoanodes for Efficient Solar Water Splitting. *Sci. Rep.* **2013**, *3* (1), 2681. https://doi.org/10.1038/srep02681.

(13) Ngamou, P. H. T.; Bahlawane, N. Influence of the Arrangement of the Octahedrally Coordinated Trivalent Cobalt Cations on the Electrical Charge Transport and Surface Reactivity. *Chem. Mater.* **2010**, *22* (14), 4158–4165. https://doi.org/10.1021/cm1004642.

(14) Cheng, C.-S.; Serizawa, M.; Sakata, H.; Hirayama, T. Electrical Conductivity of Co3O4 Films Prepared by Chemical Vapour Deposition. *Mater. Chem. Phys.* **1998**, *53* (3), 225–230. https://doi.org/10.1016/S0254-0584(98)00044-3.

(15) Cao, H.; Qiu, X.; Liang, Y.; Zhao, M.; Zhu, Q. Sol-Gel Synthesis and Photoluminescence of p-Type Semiconductor Cr2O3 Nanowires. *Appl. Phys. Lett.* **2006**, *88* (24), 241112. https://doi.org/10.1063/1.2213204.

(16) Cheng, C.-S.; Gomi, H.; Sakata, H. Electrical and Optical Properties of Cr2O3 Films Prepared by Chemical Vapour Deposition. *Phys. Status Solidi A* **1996**, *155* (2), 417–425. https://doi.org/10.1002/pssa.2211550215.

(17) O'Donnell, K. P.; Chen, X. Temperature Dependence of Semiconductor Band Gaps. *Appl. Phys. Lett.* **1991**, *58* (25), 2924–2926. https://doi.org/10.1063/1.104723.

(18) Manoogian, A.; Woolley, J. C. Temperature Dependence of the Energy Gap in Semiconductors. *Can. J. Phys.* **1984**. https://doi.org/10.1139/p84-043.
(19) Manoogian, A.; Leclerc, A. Determination of the Dilation and Vibrational Contributions to the Energy Band Gaps in Germanium and Silicon. *Phys. Status Solidi B* **1979**, *92* (1), K23–K27. https://doi.org/10.1002/pssb.2220920147.
(20) Godin, R.; Wang, Y.; Zwijnenburg, M. A.; Tang, J.; Durrant, J. R. Time-Resolved Spectroscopic Investigation of Charge Trapping in Carbon Nitrides Photocatalysts for Hydrogen Generation. *J. Am. Chem. Soc.* **2017**, *139* (14), 5216–5224. https://doi.org/10.1021/jacs.7b01547.
(21) Hayes, D.; Hadt, R. G.; Emery, J. D.; Cordones, A. A.; Martinson, A. B. F.; Shelby, M. L.; Fransted, K. A.; Dahlberg, P. D.; Hong, J.; Zhang, X.; Kong, Q.; Schoenlein, R. W.; Chen, L. X. Electronic and Nuclear Contributions to Time-Resolved Optical and X-Ray Absorption Spectra of Hematite and Insights into Photoelectrochemical Performance. *Energy Env. Sci* **2016**, *9* (12), 3754–3769. https://doi.org/10.1039/C6EE02266A.
(22) Forster, M.; Cheung, D. W. F.; Gardner, A. M.; Cowan, A. J. Potential and Pitfalls: On the Use of Transient Absorption Spectroscopy for in Situ and Operando Studies of Photoelectrodes. *J. Chem. Phys.* **2020**, *153* (15), 150901. https://doi.org/10.1063/5.0022138.
(23) Zhang, J.; Lin, Q.; Wang, Z.; Liu, H.; Zhang, Y. Identifying the Spectroelectrochemical Characteristics of Hematite Photoanodes for Water Oxidation. **2021**. https://doi.org/10.26434/chemrxiv.14504955.v1.
(24) Selim, S.; Pastor, E.; García-Tecedor, M.; Morris, M. R.; Francàs, L.; Sachs, M.; Moss, B.; Corby, S.; Mesa, C. A.; Gimenez, S.; Kafizas, A.; Bakulin, A. A.; Durrant, J. R. Impact of Oxygen Vacancy Occupancy on Charge Carrier Dynamics in BiVO4 Photoanodes. *J. Am. Chem. Soc.* **2019**, *141* (47), 18791–18798. https://doi.org/10.1021/jacs.9b09056.
(25) van Dijken, A.; Meulenkamp, E. A.; Vanmaekelbergh, D.; Meijerink, A. The Kinetics of the Radiative and Nonradiative Processes in Nanocrystalline ZnO Particles upon Photoexcitation. *J. Phys. Chem. B* **2000**, *104* (8), 1715–1723. https://doi.org/10.1021/jp993327z.
(26) Cohn, A. W.; Janßen, N.; Mayer, J. M.; Gamelin, D. R. Photocharging ZnO Nanocrystals: Picosecond Hole Capture, Electron Accumulation, and Auger Recombination. *J. Phys. Chem. C* **2012**, *116* (38), 20633–20642. https://doi.org/10.1021/jp3075942.
(27) Fan, Y.; Lin, Y.; Wang, K.; Zhang, K. H. L.; Yang, Y. Intrinsic Polaronic Photocarrier Dynamics in Hematite. *Phys. Rev. B* **2021**, *103* (8), 085206. https://doi.org/10.1103/PhysRevB.103.085206.
(28) Pendlebury, S. R.; Wang, X.; Le Formal, F.; Cornuz, M.; Kafizas, A.; Tilley, S. D.; Grätzel, M.; Durrant, J. R. Ultrafast Charge Carrier Recombination and Trapping in Hematite Photoanodes under Applied Bias. *J. Am. Chem. Soc.* **2014**, *136* (28), 9854–9857. https://doi.org/10.1021/ja504473e.
(29) Cooper, J. K.; Reyes-Lillo, S. E.; Hess, L. H.; Jiang, C.-M.; Neaton, J. B.; Sharp, I. D. Physical Origins of the Transient Absorption Spectra and Dynamics in Thin-Film Semiconductors: The Case of BiVO 4. *J. Phys. Chem. C* **2018**, *122* (36), 20642–20652. https://doi.org/10.1021/acs.jpcc.8b06645.
